# Effects of adding aerobic physical activity to strengthening exercise on hip osteoarthritis symptoms: protocol for the PHOENIX randomised controlled trial

**DOI:** 10.1186/s12891-022-05282-0

**Published:** 2022-04-18

**Authors:** Michelle Hall, Kim Allison, Rana S. Hinman, Kim L. Bennell, Libby Spiers, Gabrielle Knox, Melanie Plinsinga, David M. Klyne, Fiona McManus, Karen E. Lamb, Ricardo Da Costa, Nicholas J. Murphy, Fiona L. Dobson

**Affiliations:** 1 Centre for Health, Exercise and Sports Medicine, Department of Physiotherapy, University of Melbourne, Melbourne, Victoria 3010 Australia; 2 Menzies Health Institute Queensland, Griffith University, Brisbane & Gold Coast, Australia; 3 NHMRC Centre of Clinical Research Excellence in Spinal Pain, Injury and Health, School of Health and Rehabilitation Sciences, The University of Queensland, Brisbane, QLD 4072 Australia; 4 Centre for Epidemiology and Biostatistics, Melbourne School of Population and Global Health, The University of Melbourne, Melbourne, Victoria Australia; 5 MISCH (Methods and Implementation Support for Clinical Health research platform), Faculty of Medicine, Dentistry and Health Sciences, The University of Melbourne, Melbourne, Victoria Australia; 6 Be Active, Sleep, Eat Facility, Department of Nutrition, Dietetics and Food, Monash University, Melbourne, Australia; 7 Department of Orthopaedic Surgery, John Hunter Hospital, Newcastle, Australia; 8 Kolling Institute of Medical Research, Institute of Bone and Joint Research, University of Sydney, Sydney, Australia

**Keywords:** Osteoarthritis, Exercise, Hip, Pain, Physical function, Physical activity, Aerobic, Strengthening, Clinical trial

## Abstract

**Background:**

Hip osteoarthritis (OA) is a leading cause of musculoskeletal pain. Exercise is a core recommended treatment. Most evidence is based on muscle-strengthening exercise, but aerobic physical activity has potential to enhance clinical benefits. The primary aim of this study is to test the hypothesis that adding aerobic physical activity to a muscle strengthening exercise leads to significantly greater reduction in hip pain and improvements in physical function, compared to a lower-limb muscle strengthening exercise program alone at 3 months.

**Methods:**

This is a superiority, 2-group, parallel randomised controlled trial including 196 people with symptomatic hip OA from the community. Following baseline assessment, participants are randomly allocated to receive either i) aerobic physical activity and muscle strengthening exercise or; ii) muscle strengthening exercise only. Participants in both groups receive 9 consultations with a physiotherapist over 3 months. Both groups receive a progressive muscle strengthening exercise program in addition to advice about OA management. The aerobic physical activity plan includes a prescription of moderate intensity aerobic physical activity with a goal of attaining 150 min per week. Primary outcomes are self-reported hip pain assessed on an 11-point numeric rating scale (0 = ‘no pain’ and 10 = ‘worst pain possible’) and self-reported physical function (Western Ontario and McMaster Universities Osteoarthritis Index physical function subscale) at 3 months. Secondary outcomes include other measures of self-reported pain (assessed at 0, 3, 9 months), self-reported physical function (assessed at 0, 3, 9 months), performance-based physical function (assessed at 0, 3 months), joint stiffness (assessed at 0, 3, 9 months), quality of life (assessed at 0, 3, 9 months), muscle strength (assessed at 0, 3 months), and cardiorespiratory fitness (assessed at 0, 3 months). Other measures include adverse events, co-interventions, and adherence. Measures of body composition, serum inflammatory biomarkers, quantitative sensory measures, anxiety, depression, fear of movement and self-efficacy are included to explore causal mechanisms.

**Discussion:**

Findings will assist to provide an evidence-based recommendation regarding the additional effect of aerobic physical activity to lower-limb muscle strengthening on hip OA pain and physical function.

**Trial registration:**

Australian New Zealand Clinical Trials Registry reference: ACTRN 12619001297112. Registered 20th September 2019.

**Supplementary Information:**

The online version contains supplementary material available at 10.1186/s12891-022-05282-0.

## Background

Osteoarthritis (OA) is the 11th leading cause of disability worldwide [1] and hip OA affects one in four adults over their lifetime [2]. In Australia, arthritis-related health care costs exceed $2.1 billion AUD annually, of which OA is the largest contributor [3]. The greatest driver of health care costs for hip OA is joint replacement surgery. The lifetime risk of hip replacement for hip OA in the population is up to 12.6% [4]. Treatments that reduce symptoms and delay the need for joint replacement are critical. Current OA clinical guidelines emphasise that non-drug, non-surgical strategies [5–7] are the core management strategies for hip OA and should be offered prior to consideration for surgical management.

A 2017 meta-analysis of land-based exercise randomised controlled trials (RCTs) in hip OA identified 12 eligible RCTs and reported small-to-modest beneficial effects of exercise on pain (effect size − 0.24, 95%CI: − 0.42, − 0.06) and physical function (effect size − 0.34, 95%CI: − 0.50, − 0.18) compared to no exercise [8, 9]. Of note, all trials in the systematic review evaluated lower-limb muscle strengthening exercise, while only 3 investigated aerobic physical activity. Thus, current advice advocating exercise for hip OA is predominantly based on lower-limb strengthening interventions, which may account for the reported small-to-modest beneficial effects of land-based exercise on hip OA symptoms. Hip and thigh muscle weakness is widely established in people with hip OA [10]. However, muscle strengthening exercise in isolation likely inadequately alleviates pain and physical dysfunction in many people with hip OA. In 195 people with hip OA, we found very large and unfeasible increases in muscle strength are probably required to achieve clinically meaningful improvements in symptoms for many patients [11] – highlighting the limitation of muscle strengthening alone to improve hip OA symptoms.

Many people with hip OA do not meet aerobic physical activity guidelines [12], defined by The World Health Organisation as at least 150 min of moderate-intensity aerobic physical activity or at least 75 min of vigorous-intensity aerobic physical activity throughout the week [13]. It is therefore perhaps unsurprising that people with hip OA have lower cardiovascular fitness [14], higher levels of depression and stress [15], and more often have overweight or obesity [16] compared to those without hip OA. Of concern, cardiovascular disease [17], depressive symptoms [18, 19], and obesity [18, 19] are associated with functional decline in hip and knee OA populations. Aerobic exercise, with or without strengthening exercise, improves cardiovascular fitness and psychological well-being and reduces fat mass compared to strengthening exercise alone. In a hip OA RCT, aerobic exercise had greater beneficial effects on cardiovascular fitness, overall mental health and self-efficacy related to OA symptoms compared to strengthening alone [20]. Moreover, evidence from clinical trials in healthy adults [21] and dieting adults with obesity [22], has demonstrated superior beneficial effects of a combined aerobic and strengthening exercise program on cardiovascular fitness [22], mental health [22] and fat mass [21], compared with strengthening exercise alone. However, there are no trials in hip OA evaluating the addition of aerobic exercise to a strengthening exercise program, compared to a strengthening program alone.

The primary aim of this study is to test the hypothesis that adding aerobic physical activity to a lower-limb muscle strengthening exercise program leads to significantly greater improvements in hip pain and physical function at 3 months, compared to a lower-limb muscle strengthening exercise program alone.

## Methods/design

### Trial design

This protocol is described according to the SPIRIT guidelines [23] (see Additional File 1). The trial is designed as a superiority, two-group, randomised controlled trial. Ethical approval has been obtained from the University of Melbourne Human Ethics Committee (HREC 12710). The trial is prospectively registered in the Australian New Zealand Clinical Trial Registry (ACTRN 12619001297112). The current study protocol is available online as Additional File 2 with details of amendments to date in Additional File 3. Due to the expected low risk of harm, a data safety monitoring committee is not deemed necessary. There is also no planned interim analysis or stopping guidelines. Nested investigation of participants’ experiences will be used to study motivation to exercise. However, this will be reported separately to the main trial results.

### Participants

We will recruit 196 participants with a clinical diagnosis of hip OA from metropolitan Melbourne, Australia using community advertisements, social media, media campaigns, and our research volunteer databases. Hip OA is classified according to the National Institute for Health and Care Excellence clinical criteria for OA [24].

Participants are included if they:

i) are aged ≥45 years;
ii) have activity-related hip joint pain;
iii) no hip joint morning stiffness or morning stiffness ≤30 min;
iv) report history of hip pain > 3 months;
v) report hip pain on most days of the past month;
vi) report an average hip pain over the past week of at least 4 on an 11-point numerical rating scale (NRS, with terminal descriptors of ‘no pain’ (0) and ‘worst pain possible’ (10));
vii) pass the American College of Sports Medicine Exercise pre-participation Health Screening Questionnaire [25]; or in cases of failure, clearance must be obtained from the participant’s general practitioner to participate in this study
viii) have access to a device with internet connection.

Participants are excluded if they:

i) are on a waiting list or planning back/lower limb surgery in the next 12 months;
ii) have had previous hip replacement in the affected hip;
iii) have undergone any hip surgery in the past 6 months;
iv) are currently taking corticosteroids or have done so in the past 3 months;
v) have had any hip injections in the past 3 months or planned injection in the next 9 months;
vi) are participating in a strengthening exercise program at least 3 times per week and/or engaging in 150 min of moderate intensity aerobic physical activity per week within the past 6 months;
vii) have self-reported inflammatory arthritis;
viii) have any neurological condition affecting lower limb and ability to exercise safely;
ix) have any unstable or uncontrolled cardiovascular condition;
x) are unable or unwilling to comply with the study protocol;
xi) are unable to speak and/or read English;
xii) are pregnant or planning pregnancy.

### Data collection and management

**Fig. 1:**
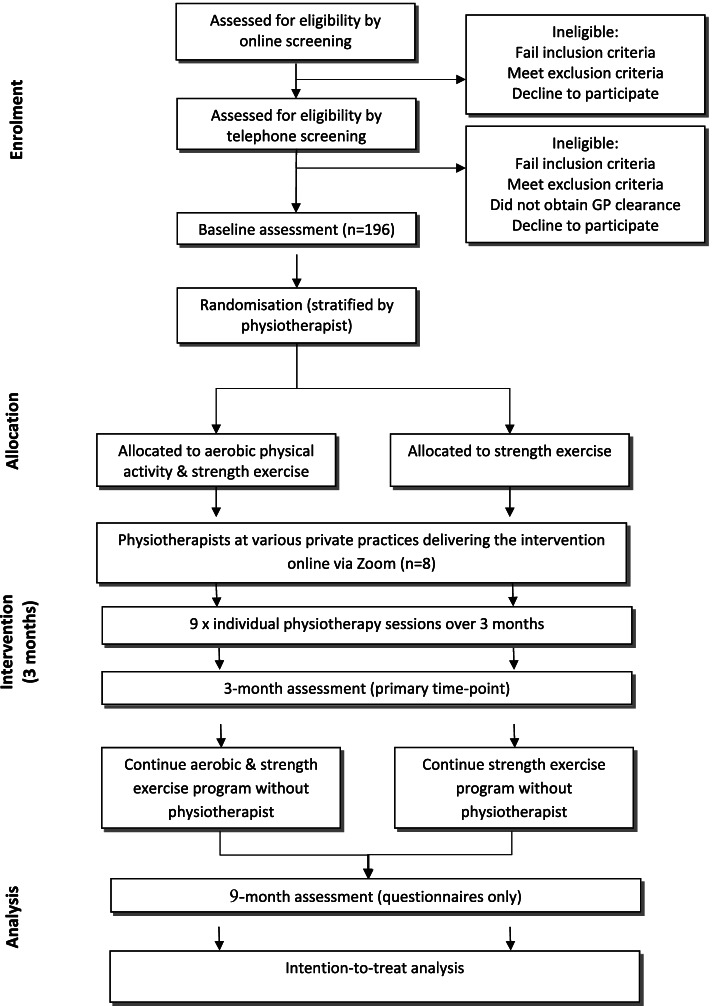
Participant flow through the randomised controlled trial

Figure 1 outlines the phases of the trial. Volunteers are screened online, then over the telephone. Participants receive a detailed verbal description of the project to ensure that the trial procedures are understood during the telephone screening process. If participants pass the telephone screening process, participants are sent the Plain Language Statement, and Consent Form (see Additional File 4) in the post or by email. After reading the Plain Language Statement, and if they give their consent to participate, written consent is acquired either online via REDCap or on paper via post using a reply-paid envelope or email to the Trial Coordinator. Screening information and study consent forms are stored within a secure data collection platform (Qualtrics or REDCap) and accessible only by password to the researchers. Participants are then scheduled to attend 1) the Centre for Health, Exercise and Sports Medicine at The University of Melbourne for performance-based physical function, strength measures and quantitative sensory testing, 2) the Be Active Eat Sleep (BASE) facility at Monash University for cardiorespiratory fitness and body composition assessment, and 3) one of the study radiology clinics for a supine anteroposterior hip x-ray. Participants who provide the researchers with a weight bearing or supine anteroposterior hip x-ray obtained in the past 12 months do not undergo a new x-ray due to ethical concerns of exposing them to additional radiation. Blood sampling is an opt-in assessment where participants are invited to provide blood samples at one of the study pathology clinics. For participants with bilaterally eligible hips, the most symptomatic hip as identified by the participant is evaluated.

Paper-based or electronic questionnaires are sent to participants at baseline, 3 months and 9 months to complete self-reported primary and self-reported secondary outcomes. At the 3-month follow-up, participants return to the University of Melbourne and Monash University for re-assessment of performance-based physical function, strength, cardiorespiratory fitness, quantitative sensory measures and body composition. Participants who provided a blood sample at baseline are asked to attend a study pathology clinic to provide a follow-up blood sample. Participants also complete a logbook to record adherence to the exercise program over the first 3 months. If questionnaires or logbooks are not returned, the participants are contacted to prompt a response, or as a last resort, to obtain primary outcome data via telephone or email.

### Randomisation and allocation concealment

Following baseline assessments, participants are randomised to one of 8 study physiotherapists using a block randomisation list prepared by an independent statistician. If a physiotherapist is unavailable, participants are re-randomised to another available physiotherapist. Once allocated to a physiotherapist, participants are allocated to a treatment group using a randomisation schedule prepared by an independent biostatistician according to a 1:1 allocation in blocks of varying sizes stratified by physiotherapist. The randomisation schedule is stored on a password-protected website (REDCap™) maintained by a researcher not involved in either participant recruitment or administration of the outcome measures. Each participant receives a unique study ID code, and this is documented in the participant’s record with the study database in addition to all study documents. Group allocation is revealed by staff with no other involvement in the study upon completion of baseline assessments.

Participants are blinded to group allocation by a process of limited disclosure. Participants are informed that the study is evaluating two different undisclosed ‘types’ of exercise. Participants are not told about the types of exercise under investigation. Participants are not informed about the study hypotheses, or which group they are allocated to until the study is completed. Only when the study is completed will participants be provided with a lay summary of the study purpose, hypotheses and findings. The primary outcomes and some of the secondary outcomes are participant-reported, so participants are also the unblinded assessors. Staff conducting assessments for some of the secondary outcomes (e.g. body composition) are blinded. The physiotherapists are unblinded to group allocation or hypothesis. Statistical analyses will be performed blinded.

### Interventions

Interventions have been described according to the TIDIeR checklist [26] and resources provided to participants are described in Table 1. Eight experienced physiotherapists in private practice in Melbourne, Australia who have treated at least five individuals with hip OA in the past 12 months underwent training to deliver the interventions. The physiotherapists were trained to deliver both exercise programs. Training comprised of a 4-hour workshop conducted by musculoskeletal physiotherapists on the research team. The physiotherapists were provided with a detailed study manual, in addition to customised study treatment notes.

**Table 1:** Summary of resources provided to participants by group allocation

Resource	Description	Aerobic physical activity and strengthening group	Strengthening exercise only group
Consultations with a physiotherapist	9-video consultations over 3-months. Physiotherapist provides structured strengthening exercise plan and behaviour change support.		□
	9-video consultations over 3-months. Physiotherapist provides structured strengthening exercise and physical activity plan and behaviour change support.	□	
Exercise bands	4 exercise resistance bands (yellow, red, green, blue) for strengthening exercises	□	□
Exercise weights	Ankle cuff weight	□	□
Exercise mats Activity monitor	Exercise mat Garmin Vivosmart 4 provided for 3-months	□	□
**Booklets**			
Preparing for your consultations	Details about consultations, instruction on how to use Zoom videoconferencing	□	□
Osteoarthritis information	Information about osteoarthritis, overcoming exercise and activity barriers, strengthening exercise for managing hip pain and managing a flare-up of hip pain	□	□
Exercise booklet	Strengthening exercise instructions and photographs	□	□
Strengthening exercise logbook	Logbook to record details of management plans and complete exercises		□
Strengthening exercise and aerobic physical activity logbook	Logbook to record details of management plans and complete exercises and physical activity	□	
Garmin instructions booklet	Detailed instructions on how to use the Garmin Vivosmart 4	□	□

*OA* Osteoarthritis

All participants are asked to attend 9 physiotherapist consultations over 12 weeks: once per week in the first 6 weeks and approximately once every 2 weeks thereafter. The initial session is 60 min for the combined aerobic and strengthening exercise group and 45 min for the strengthening exercise only group to enable adequate coverage of the study procedures, patient materials and initial customisation of either the strength only or combined strength and aerobic exercise programs. The remainder of the sessions is 20 min for the strengthening only exercise group, and 30 min for the combined strengthening and aerobic exercise group. Interventions are delivered individually in a one-on-one consultation via videoconference (e.g. Zoom). Exercise dosage (i.e. frequency, intensity, time) is consistent with the American College of Sports Medicine (ACSM) recommendations [27] as described below for each exercise program. The ACSM recommendations were used based on a meta-analysis demonstrating that exercise programs prescribed in accordance with ACSM dosage recommendations resulted in greater symptom improvement compared to exercise programs with questionable concordance to ACSM dosage recommendations [8, 9].

Participants are provided with a wrist worn activity monitor (Garmin Vivosmart 4) at baseline and are asked to continuously wear the monitor throughout the 3-month program. Heart rate accuracy during various types of exercise at various intensities has been previously reported^28^. During baseline assessment, participants are asked to walk until their rating of perceived exertion (RPE) on the Borg Scale [28] reaches “somewhat hard”. Researchers record the heart rate per minute when participants reach “somewhat hard” RPE. For participants in the combined aerobic physical activity and strengthening exercise group, aerobic exercise intensity is prescribed at this RPE and heart rate level. The number of minutes participants spend above desired heart rate is obtained per week by the research team using the Fitabase platform (Small Steps Lab LLC).

#### Education material and self-management support

All participants receive booklets containing information about OA (Table 1), overcoming exercise and physical activity barriers, strengthening exercise for management of hip pain and managing pain. Physiotherapists discuss pain from a biopsychosocial perspective and participants are advised that it is acceptable and safe to experience moderate levels of pain during exercise. This is based on evidence from a systematic review and meta-analysis demonstrating that protocols allowing painful exercises yielded a greater beneficial effect, albeit small, compared to studies that prescribed pain free exercises for chronic musculoskeletal pain in the short-term [29].

Over the initial 6 weeks of each program, physiotherapists focus closely on understanding participant beliefs surrounding pain, OA and exercise for OA symptoms. Behaviour change support applicable to exercise [30, 31] such as challenging unhelpful beliefs, advice on suitable levels of pain and on exercise modification are embedded into the consultations and resources (Table 2). During the second 6 weeks of each program, physiotherapists increase their focus on helping consolidate participant’s self-management skills and increasing their capacity and autonomy to monitor their response to exercise, progressions and modifications to their exercise program and develop a plan to support continuation of the exercise program after the physiotherapist intervention ceases at 3 months.

**Table 2:** Summary of integration of behaviour change techniques to support exercise within intervention and resources

Behaviour change technique	Written information	Physiotherapist discussion	Other
**1. Consequences of behaviour**			
Explanation of benefits of strengthening exercise ^(AS, S)^ and aerobic physical activity ^(AS)^	✓ Osteoarthritis information booklet	✓ Session 1 ✓ As dictated by patient Sessions 2–9 with checks on understanding	✓ Telephone screening ✓ Baseline assessment
Explanation that exercise & physical activity will not worsen joint structural damage ^(AS, S)^	✓ Osteoarthritis information booklet	✓ Session 1 ✓ As dictated by patient sessions 2–9 with checks on understanding	✓ Telephone screening
**2. Goal setting**			
Development of specific goals related to patient’s hip problems (goal setting – outcome) ^(AS, S)^		✓ Session 1	
Development of specific exercise goals (goal setting – behaviour) ^(AS, S)^		✓ Sessions 2–9	
**3. Action planning**			
Use of a plan stating how often to exercise & which exercises to do (including dosage) ^(AS, S)^	✓ Exercise logbook	✓ Each session	
**4. Barrier identification/problem solving**			
Information & discussion about barriers to exercise ^(AS, S)^ & physical activity ^(AS)^ adherence, including problem-solving	✓ Osteoarthritis information booklet	✓ Each session, transition from barriers and facilitators to self-management/regulation in the absence of physiotherapy contact in the final 3 sessions	
**5. Behavioural grading**			
Strengthening exercises are graded in number, intensity and/or difficulty to get progressively harder over time. ^(AS, S)^	✓ Osteoarthritis information booklet ✓ Exercise logbook	✓ Each session	✓ Four graded resistance bands ✓ Adjustable ankle-cuff weights
**6. Instruction & information**			
Instruction in where, when and how to perform strengthening exercises. ^(AS, S)^	✓ Exercise logbook ✓ Exercise booklet	✓ Each session	
Demonstration of how to perform strengthening exercises. ^(AS, S)^	✓ Exercise booklet	✓ Each session	
Demonstration of how to reach desired heart rate intensity for aerobic exercise ^(AS)^			✓ Telephone consult with Trial Coordinator +/− during lab baseline assessment for those who attend
**7. Self-monitoring & feedback**			
Encouraged to self-monitor strengthening exercise ^(AS, S)^ and aerobic exercise ^(AS)^ intensity	✓ Exercise logbook ✓ Exercise booklet	Each session	✓ Garmin provided and instructions to combined group to monitor heart rate
Encouraged to record strengthening ^(AS, S)^ and aerobic ^(AS)^ completed exercises	✓ Exercise logbook	✓ Each session	
Physiotherapist review of & and feedback on exercise recorded.		✓ Ongoing throughout program, greater focus weeks 1–6	
**8. Relapse prevention**			
Instruction on how to modify exercise & physical activity during flare-ups. ^(AS, S)^		✓ Each session	
Planning for setbacks in physical activity & how to overcome them. ^(AS)^		✓ Ongoing throughout physiotherapy sessions, strong focus final 3 sessions	
Dealing with lapses & setbacks with exercise & physical activity; use of constructive self-talk. ^(AS, S)^		✓ Ongoing throughout physiotherapy sessions, strong focus final 3 sessions	
**9. Emotional control**			
Encouraged to use activity pacing & pain coping activities	✓ Osteoarthritis information booklet	✓ Each session, as indicated by participant	
Encouraged to use pain coping strategies.	✓ Osteoarthritis information booklet		
**10. Prompts**			
Encouraged to use reminders to exercise.	✓ Exercise logbook		
**11. Rewards**			
Physiotherapist congratulates adherence to exercise.		✓ Ongoing throughout physiotherapy sessions, specific mid-point review of goals at six weeks	
Options discussed to use self-rewards for attempts towards goals as well as positive reinforcement towards achieving goals		✓ Session 7, reflection on strategies for positive reinforcement	
**12. Social support and accountability**			
Encouraged to involve partner or family to join in with exercising and beyond social accountability to physiotherapy		✓ Session 7, discussion to shift social accountability from physiotherapy	
**13. Review**			
Review, supervision and correction of strengthening exercise technique.		✓ Each session	
Review of tracked minutes of aerobic physical activity ^(AS)^		✓ Each session	✓ Garmin tracking application
Review of outcome goals at follow-up.		✓ Session 7	

^(AS)^ Combined strengthening and aerobic physical activity group

^(S)^ Strengthening exercise only group

#### Strengthening exercise program

The purpose of the strengthening program is to enhance lower limb muscle strength. The physiotherapists instruct participants on how to perform 4–6 lower limb strengthening exercises (see Additional File 5), including how to use equipment (free weights and exercise resistance bands). Initial sessions involve 1 to 2 sets of 8 to 12 repetitions of at least “hard” (≥7) on a rating of perceived exertion modified (0–10) Borg Scale, increasing as appropriate to 3 sets at “hard to very hard” (between 7 to 8) intensity. Emphasis is placed on the intensity of the exercise (RPE) rather than volume (repetitions and sets). Strengthening exercises are to be completed 3 times per week at home and take approximately 20 min per session.

#### Aerobic physical activity and strengthening exercise program

In addition to the aforementioned lower-limb strengthening program, participants receive an aerobic physical activity program. The purpose of adding aerobic physical activity to strengthening exercise is to improve cardiovascular fitness, psychological well-being and body composition. Despite the paucity of research in hip OA, evidence from people with knee OA supports a graded relationship between increased moderate-to-vigorous physical activity and reduced disability [32]. The greatest reductions in disability were noted when increasing moderate-to-vigorous activity to guideline levels [32]. Hence, physiotherapists facilitate participants to build up to engaging in 150 min of moderate intensity aerobic physical activity per week.

We refer to the aerobic component of our trial as ‘aerobic physical activity’ given this included planned aerobic exercise, as well as incidental aerobic activity. Participant’s individual preference determines the type of aerobic physical activity, which may be a combination of more than one type of aerobic exercise (e.g. walking and cycling). Participants are encouraged to incorporate aerobic physical activity into their daily lives and guided to gradually increase their aerobic physical activity at moderate intensity by at least 10-min blocks per week over the 3-month supervised intervention period until 150 min of at least moderate intensity exercise per week is achieved. Physiotherapists guide participants to self-manage their aerobic physical activity at an intensity equivalent to at least “somewhat hard” (≥13) on a RPE Borg Scale (6–20) and personalised corresponding heart rate on their wearable activity monitor.

### Outcome measures

Table 3 summarises the outcome measures. Validated measures of pain and physical function that have been recommended for use in hip OA clinical trials are used for primary outcomes [33]. Treatment efficacy will be based on the 3-month changes in our primary outcomes.

**Table 3:** Summary of measures

	Data collection instrument	Time-points		
		Baseline	3 mths	9 mths
**Descriptive data**				
Age, sex, body mass index, education level, current employment status		□		
Duration of hip OA symptoms, laterality of symptoms		□		
Comorbidities	Self-Administered Comorbidity Questionnaire	□		
Radiographic disease severity	Standard supine AP pelvis x-ray	□		
Expectation of treatment outcome	5-point ordinal scale	□		
Therapeutic alliance^a^	Working Alliance Inventory-Short Revised questionnaire			
**Primary outcomes**				
Overall average hip pain in the past week	11-point NRS	□	□	□
Physical function in past 48 h	WOMAC physical function subscale	□	□	□
**Secondary outcomes**				
Pain	WOMAC pain subscale	□	□	□
	Hip pain during walking, NRS	□	□	□
Stiffness	WOMAC stiffness subscale	□	□	□
Perceived change since baseline	Change in pain, 7-point ordinal scale		□	□
	Change in function, 7-point ordinal scale		□	□
Health-related quality of life	AQoL-6D questionnaire	□	□	□
Muscle strength	Hip extensor strength	□	□	
	Hip abductor strength	□	□	
	Knee extensor	□	□	
Physical function	Patient specific functional scale	□	□	□
	30 s sit to stand	□	□	
	Timed stair climb	□	□	
	40 m fast walk	□	□	
Cardiorespiratory fitness	Submaximal cardiorespiratory fitness	□	□	
**Other measures**				
Adherence	Number of consultations with physiotherapist		□	
	Number of strength sessions at least “very hard”(RPE)		□	
	Number of activity minutes at minimum prescribed heart rate		□	
Treatment fidelity	Number of exercise prescriptions according to protocol		□	
Adverse events	Number and nature (related, non-related, severity)		□	□
Co-interventions	Number and type	□	□	□
Satisfaction with exercise programs	11-point NRS		□	
**Mechanistic measures**	Body composition (fat mass, lean mass)	□	□	
	Inflammatory makers (e.g. IL-6, TNF- α, CRP)	□	□	
	Quantitative sensory measures (PPT, TS, CPM)	□	□	
	Depression, anxiety, stress (DASS-21 subscale)	□	□	□
	Neuropathic pain (PainDETECT)	□	□	□
	Self-efficacy for walking (SEWS-D, mGES)	□	□	□
	Fear of movement (BFMS)	□	□	□
	Sleep quality (PSQI)	□	□	□
	Fatigue (MDAF)	□	□	□

*NRS* Numeric rating scale, *WOMAC* Western Ontario and McMaster Universities Osteoarthritis Index, *AQoL-6D* Assessment of Quality of Life Instrument, *RPE* Rate of perceived exertion, *IL-6* Include interleukin-6 TNF- α = tumor necrosis factor (TNF-α), and C-reactive protein, *PPT* Pain pressure threshold, *TS* Temporal summation, *CPM* Condition pain modulation, *DASS* Depression, Anxiety, and Stress Scale, *SEWS-D* Self-efficacy for Walking Scale – Duration, *mGES* Modified Gait Efficacy Scale, *BFMS* Brief Fear of Movement Scale for osteoarthritis, *PSQI* Pittsburgh Sleep Quality Index, *MDAF* Multi-Dimensional Assessment of Fatigue, *RPE* Rate of perceived exertion

^a^also recorded at 6 weeks

#### Primary outcomes

##### Severity of overall average hip pain scored on an 11-point NRS

At baseline, 3 (primary time point) and 9 months, overall average hip pain intensity in the last week is self-reported using an 11-point NRS. Scores range from 0 to 10, where 0 represents ‘no pain’ and 10 represents the ‘worst pain possible’. This outcome has demonstrated reliability and validity in OA [34] with reported minimal clinically important difference (MCID) of 1.8 out of 10 for NRS pain [35].

##### Physical function subscale of the WOMAC

At baseline, 3 (primary time point) and 9 months, difficulty with physical function is assessed by the Western Ontario and McMaster Universities (WOMAC) Osteoarthritis Index (Likert version 3.1) [36]. The physical function subscale contains 17 questions each answered on a Likert Scale where 0 is ‘no difficulty’ and 4 is ‘extreme difficulty’. Scores range between 0 to 68, with higher scores indicating greater difficulty with physical dysfunction. This outcome has also demonstrated reliability and validity in OA [36] with a reported MCID of 6 out of 68 points for WOMAC function [37].

#### Secondary outcomes

##### Stiffness subscale of the WOMAC

At baseline, 3 and 9 months, movement stiffness is assessed using the 2-item stiffness subscale of the WOMAC, with Likert response options ranging from 0 being ‘no stiffness’ to 4 being ‘extreme stiffness’. Scores range from 0 to 8, higher scores indicating greater stiffness [36].

##### Pain subscale of the WOMAC

At baseline, 3 and 9 months, pain is assessed using the 5-item pain subscale of the WOMAC, with Likert response options ranging from 0 being ‘no pain’ to 4 being ‘extreme pain’. Scores range from 0 to 20, higher scores indicating greater pain [36].

##### Severity of overall average hip pain during walking

At baseline, 3 and 9 months, overall average hip pain during walking in the last week is self-reported using an 11-point NRS. Scores range from 0 to 10, where 0 represents ‘no pain’ and 10 represents the ‘worst pain possible’ [34].

##### Participant-perceived global ratings of change

At 3 and 9 months, participants rate perceived change in i) hip pain and ii) physical function since starting the study, and over the past 6 months, respectively. Response to treatment is scored using a 7-point Likert scale from ‘much worse’ to ‘much better’. Participants who report ‘moderately better’ or ‘much better’ are classified as ‘improved’ and all other respondents are classified as ‘not improved’ [38].

##### Patient specific functional scale

At baseline, participants are asked to indicate up to five activities of daily living in which performance was limited in the previous week and rate these on an 11-point NRS ranging from 0 to 10, where 0 indicates ‘unable to perform’ and 10 indicates ‘able to perform at the same level as before injury or problem’. Total score is the sum of the ratings for each activity divided by the number of activities, with higher scores indicating better function [39]. At 3 and 9 months, participants are be asked about the activities they listed at baseline and provide a rating on the same scale.

##### Health related quality of life

At baseline, 3 and 9 months, health-related quality of life are be measured by the Assessment of Quality of Life (AQoL) [40] (version AQoL-6D). It consists of 20 items that assess independent living, mental health, relationships, pain, coping and senses. Scores range from − 0.04 to 1.00, with higher scores indicating better quality of life.

##### Muscle strength

At baseline and 3 months, maximal normalised isometric hip abduction, hip extension and knee extensor strength (peak torque, Nm/kg) is measured in accordance with previously described reliable procedures [41, 42]. Maximal isometric hip abductor torque is measured using a hand-held dynamometer (Lafayette Instruments, IN, USA) and maximal isometric hip extensor torque at 20° hip flexion is measured using a force transducer (Shimpo Instruments, NY, USA. Measurements of hip abductors and extensors are taken in supine. After one, sub-maximal warm up trial, participants perform two maximal trials, each of 3 s in duration separated by 30 s of rest. The mean of the two trials is used for analysis. Maximum voluntary isometric torque of the quadriceps muscles at 60° knee flexion is measured in sitting using a HUMAC isokinetic dynamometer (Humac NORM, CSMI, Massachusetts, USA). After two, sub-maximal warm-up trials to familiarise participants with the testing procedure, participants perform three maximal contraction trials, each of 5 s in duration separated by 30 s of rest. The peak value of these three trials is used for analysis [41]. The sum of hip abduction, hip extensor and knee extensor strength is calculated and will be reported as an overall strength score.

##### Physical performance

At baseline and 3 months, physical performance is measured using the Osteoarthritis Research Society International set of performance-based measures of physical function, including the 30-s sit-to-stand test, 40-m fast pace walk test and 6-step stair climb test [43, 44]. For the 30-s sit-to-stand test, following a practice trial, the number of times participants can rise to a full standing position from sitting and return to sitting, with arms crossed and held against the chest, within 30 s is counted. A greater number indicates better performance. For the 40 m fast pace walk test, participants are timed (seconds) to determine how long it takes them to walk 40 m in their usual footwear with the instruction ‘walk as quickly as you can without overexerting yourself’. This is performed once and the 40 m time recorded, with less time indicating better performance. For the 6-step stair climb test, the time (seconds) to walk up and down six 17.5 cm high steps as quickly as possible using a handrail if preferred is recorded, with less time indicating better performance.

##### Cardiorespiratory fitness

At baseline and 3 months, a submaximal exercise test is performed on a cycle ergometer (Corival, Lode B.V. Groningen, The Netherlands), and submaximal cardiorespiratory fitness is measured by breath-by-breath indirect calorimetry using a Vmax Encore metabolic cart (Carefusion, San Diego, CA, USA). Procedures are adjusted from standard fitness testing protocols [45] to suit participant clinical presentation. Total and relative (kg BM) oxygen consumption ($\overset{.}{V}$O_2_ per min) are determined and will be reported. The height of the seat is individually adjusted for participant leg length and participants are asked to keep a cadence of 60–70 pedal rounds per minute. Heart rate is measured by a pulse belt (Polar Electro, Kempele, Finland) and RPE by Borg Scale [28] during the ~ 16-min assessment. Participants begin the test with an 8–10 min light warm up and start at 1 W/kg FFM and increase 0.5 W/kg FFM every 3 min until they cannot maintain the watt output at ≥60 rpm, or they reach a rating of perceived exertion (RPE) of 15–17. Data are used to establish aerobic economy as previously reported [46, 47].

#### Mechanistic measures

Mechanistic measures are being collected as part of the study to undertake exploratory mediation analyses once findings from the trial have been reported.

##### Neuropathic-related pain

At baseline, 3 and 9 months, the painDETECT questionnaire is used to determine how likely pain has a neuropathic component. The tool includes questions about hip pain intensity (NRS, 0 to 10 where higher scores indicate greater severity), pain course pattern (option from four illustrations), pain radiation (yes/no question) and somatosensory phenomena (seven questions on a 6-point Likert Scale, 0 to 5 with higher scores indicating likelihood of somatosensory phenomena). Total scores range from 0 to 38 with higher scores indicating more neuropathic-like symptoms. Scores of < 12 indicate pain is unlikely to be neuropathic and scores of > 19 suggest pain is likely to have neuropathic components [48].

##### Depression, anxiety and stress

At baseline, 3 and 9 months, emotional states of depression, anxiety and stress are measured by the 21-item Depression, Anxiety and Stress Scale (DASS) using 4-point Likert response, with options ranging from 0 (‘did not apply to me’) to 3 (‘applied to me very much, or most of the time’). Scores range from 0 to 42, with higher scores indicating greater levels of distress [49].

##### Self-efficacy for walking

At baseline, 3 and 9 months, participants’ perceived ability to walk at various durations and overcome various obstacles (e.g. stairs) is measured by the Self-Efficacy for Walking Scale – Duration [50] and the modified Gait Self-Efficacy Scale [51] respectively, using a scale ranging from 0% (not at all confident) to 100% (completely confident). Total score for each measure of self-efficacy is calculated by summing the confidence rating and dividing by the total number of items in the Scale, resulting in a maximum possible efficacy score of 100.

##### Brief fear of movement scale

At baseline, 3 and 9 months, fear of pain, movement and injury is assessed on the 6-item Brief Fear of Movement Scale [52] scored on a 4-point scale from 1 being ‘strongly disagree’ to 4 being ‘strongly agree’. The 6-item scale is scored from 6 to 24 with higher scores indicating greater fear of movement.

##### Sleep quality

At baseline, 3 months and 9 months, the Pittsburgh Sleep Quality Index (PSQI) questionnaire is used to evaluate sleep quality and fatigue, respectively over the past month. The PSQI measures seven components of sleep quality and each component is scored from 0 to 3 creating a total score from 0 to 21, with higher scores indicating poorer sleep quality [53].

##### Fatigue

At baseline, 3 and 9 months, fatigue is measured on the Multi-Dimensional Assessment of Fatigue (MAF) questionnaire. The MAF is a 16-item scale that assesses four dimensions of fatigue including degree and severity, distress, frequency and the impact of fatigue on daily living. Fifteen items are used to calculate a global fatigue index. The range of scores is 1–50, with higher scores indicating more fatigue [54].

##### Body composition

At baseline and 3 months, whole body lean mass and fat mass are assessed by iDXA (GE Lunar iDXA narrow-angle dual energy x-ray densitometer) according to the manufacturer recommendations for positioning the participant, scan protocols and scan analysis. Participants are assessed in fasted and euhydrated state (total body water measured by validated and reliability checked multifrequency bioelectrical impedance analysis; Seca 515 MBCA, Seca Group, Hamburg, Germany)]. Participants are asked to avoid strenuous exercise for a 12-h period prior to all laboratory assessments. Height is assessed using a fixed stadiometer (Holtain, Crosswell, Crymych, UK). Body mass is measured (Seca 515 MBCA) to the nearest 0.1 kg using standardized anthropometrical procedures. Total (in kilograms) and relative (in percent) fat mass (FM) and free FM is assessed by a trained radiographer.

##### Inflammatory markers

Participants can opt-in to provide ~ 8 ml blood for assessing inflammatory biomarkers at baseline and at 3-month follow-up. Those who opt provide blood samples will be instructed to fast for 12 h beforehand and not to exercise in the 12 h before samples are drawn, and to rest for 20 min before samples are drawn. Samples are stored at -70C according to the safe and secure protocol of the Clinical Trial Department at Melbourne Pathology until the end of trial when analyses will be undertaken. Biomarkers of interest such as interleukin-6 (IL-6), interleukin-1β (IL-1β), tumor necrosis factor (TNF), and C-reactive protein may be assessed using high-sensitive enzyme-linked immunosorbent assays.

##### Quantitative sensory assessments

At baseline and 3 months, somatosensory profiles are quantitatively assessed with quantitative sensory testing. Quantitative sensory measures include pressure pain threshold, temporal summation and conditioned pain modulation [55, 56]. Each test is performed at a local and remote standardised location to explore primary and secondary sensitization, respectively; 2-3 cm distal to the anterior superior iliac crest on the affected, or most affected side and 1-2 cm above the ipsilateral lateral epicondyle of the elbow.

Pressure pain threshold is assessed using a handheld pressure algometer (Somedic, Sweden), with probe size of 1cm^2^. The probe is placed on the skin and pressure is gradually increased at a rate of 30 kPa/s. The test stops when the participant feels that the sensation of pressure first changes to pain. Three repetitions with an interval of 30 s is performed and the mean value is used in analysis.

Temporal summation is measured as the perceived intensity of a single pinprick stimulus (256 Nm pinprick) compared to a series of ten repetitive pinprick stimuli of the same intensity, applied at rate of 1/s using a metronome within a 1cm^2^ area. The participant rates pain for the single pinprick and the estimated mean and worst pain for the series of 10 pinpricks on a 0 to 10 NRS is calculated. This procedure is be repeated three times. Temporal summation is calculated as the mean pain rating of the three series stimuli divided by the mean pain rating of the three single stimuli. A higher ratio indicates increased responsiveness to repeated mechanical noxious stimuli.

Conditioned pain modulation is assessed to gauge pain inhibitory control mechanisms [56]. Pressure pain threshold is measured on the standardised ipsilateral elbow (test stimulus). Participants then immerse the contralateral foot in cold water ~12C in temperature (conditioning stimulus). After 30 s of immersion, participants are asked to rate pain on a 0 to 10 NRS. Water temperature is adjusted until the participant reports a pain intensity between 4 and 6 out of 10. Once 4–6/10 pain intensity and a minimal immersion time of 30 s is reached, pressure pain threshold is measured. The conditioned pain modulation response is calculated as the difference between the test stimulus before and immediately after the conditioning stimulus [57]. A positive conditioned pain modulation reflects an analgesic response (i.e. increase in pain pressure threshold), whereas a negative conditioned pain modulation reflects a hyperalgesic response (i.e. decrease in pain pressure threshold).

#### Descriptive data

At baseline, age, sex, body mass index, education level, current employment, duration of symptoms, co-morbidities [58], radiographic disease severity [59], symptom laterality, and treatment expectation are recorded. At 6 weeks, the Working Alliance Inventory-Short Revised questionnaire [60] captures key aspects of the therapeutic alliance including a) agreement on the tasks of therapy, b) agreement on the goals of therapy and c) development of an affective bond.

#### Other measures

##### Treatment fidelity

Physiotherapists record the exercise type, dosage and intensity prescribed to participants each session in their treatment notes. We use physiotherapists’ treatment notes to determine whether in at least 7 of the 9 consultations, the following criteria were fulfilled: i) 3 strengthening exercises (from the first consultation) and 4–6 strengthening exercises (from the second consultation onwards) of at least “very hard” intensity were prescribed, and for participants in the combined aerobic physical activity and strengthening group: ii) prescribed an increase in moderate intensity aerobic physical activity by at least 10 min per week, until 150 min per week was reached. Both criteria must be satisfied for participants in the combined aerobic physical activity and strengthening group, while only the strength criteria is applicable to the strengthening only group.

##### Treatment adherence

The number of consultations with the physiotherapist is recorded. Participants record the exercise type, dosage and intensity performed in their logbooks. Participant exercise logbooks are used to determine if 3 strengthening exercises (from the first consultation) and 4–6 strengthening exercises (from the second consultation onwards) at least at a “very hard” intensity were performed, at least three times per week. Adherence for the strengthening group is considered satisfactory if participants self-report completing strengthening exercises “very hard” at least 10 weeks of the 3 months. Adherence for the combined aerobic physical activity and strengthening exercise group, is considered satisfactory if participants self-report completing strengthening exercises “very hard” and self-report completing the number of minutes at the minimum heart rate set by the physiotherapist in at least 10 weeks of the 3 months program.

##### Adverse events

Adverse events are considered as any untoward medical occurrence, irrespective of whether it is related to the study interventions or not. Participants are requested to report any adverse events to their physiotherapist. Treatment is discontinued as necessary, and further medical advice is arranged. Physiotherapists record the event in their consultation notes. The adverse events are recorded from participants at 3 and 9 months. Participants are requested to provide details on the nature of the adverse event, how long it lasted for, and what action they took, if any. The Principal Investigator along with other study investigators determine causality. If the event is related to either exercise program, it is deemed a related adverse event. Serious adverse events are considered as any untoward and unexpected medical occurrence that results in death, is life-threatening, requires hospitalisation or results in significant disability. Any serious adverse events are reported to the ethics committee.

##### Co-interventions

Use of co-interventions for hip pain and any other treatments for hip OA are self-reported at baseline, 3 months and 9 months. Participants report the frequency of use of a range of pain and arthritis medications and co-interventions.

##### Satisfaction with the exercise programs

Participants rate their satisfaction with their exercise program at 3 months using an 11-point NRS scale with terminal descriptors of ‘not at all satisfied’ to ‘extremely satisfied’.

### Data integrity

Data is maintained and stored using the REDCap™ database software using a combination of data collection and entry by researchers, and direct entry by the participants via survey links. If participants opt to complete paper-based questionnaires, there is a prompt for the researcher entering data to double-check the accuracy of the primary outcomes. The database is backed-up regularly on a secure network and is compliant with the International Council for Harmonisation Guideline for Good Clinical Practice, according to our data management plan. Study personnel are only able to access the database with a personal login and password.

### Sample size calculations

We aim to detect a MCID in change (baseline minus 3 months) in primary outcomes between groups (1.8 out of 10 for NRS pain [35] and 6 out of 68 for WOMAC function [37]). Based on data from our previous trial on exercise in hip OA [61, 62], we assume between-participant standard deviations of 2.2 for pain and 13 units for physical function, and a baseline to 3-month correlation of 0.46 for pain and 0.40 for physical function respectively. Using analysis of covariance adjusted for baseline score, and to achieve 90% power with a 5% significance level, we require 25 participants per arm to detect the MCID in between-group change in pain and 83 per arm for physical function. Allowing for 15% attrition, we will recruit 98 participants per arm for a total sample of 196 participants.

### Data analysis

A biostatistician will analyse data in a blinded manner. Main comparative analyses between groups will be performed using intention-to-treat. If more than 5% of primary outcomes are missing at 3 months (primary time point), multiple imputation will be applied as the primary analysis. Differences in mean change (baseline minus follow-up) at each timepoint (3-months and 9-months) in each primary outcome (pain and physical function) will be compared between groups separately using linear mixed-effects modelling adjusted for the outcome at baseline and physiotherapist, with random effects to account for clustering by participants. Models will include factors representing intervention, time and the intervention by time interaction. For the primary hypotheses, the absolute difference in mean change from baseline between groups will be estimated (including two-sided 95% confidence intervals) at 3 months (primary time point). Similar analyses will be conducted for continuous secondary outcomes measured at baseline, 3 and 9 months. For continuous secondary outcomes measured only at baseline and 3 months, differences in mean change in each primary outcome will be compared between groups separately using multiple linear regression adjusted for the outcome at baseline and physiotherapist. Improvement based on global change at each timepoint (3 and 9 months) will be compared between groups using risk ratios and risk differences, by fitting mixed-effects logistic regression models, adjusted for physiotherapist, with random effects to account for clustering by participants and physiotherapist. A sensitivity analysis will estimate treatment effects at 3 months assuming full adherence, using a two-stage least squares approach [63]. Standard diagnostic plots will be used to check model assumptions. A full statistical analysis plan will be published on the CHESM website prior to undertaking the formal analysis of collected data.

### Patient and public involvement

Patients with hip OA were involved in the development and how the aerobic and strengthening intervention are delivered. This multistep process included participation in the intervention (pilot), identifying acceptability of the intervention, and barriers and facilitators to participation and adherence through semi-structured telephone interviews, which were also completed by the physiotherapists delivering the intervention (in the pilot phase). Patients were also consulted on outcome measures, adaptations to intervention based on our unpublished pilot study and the plain language statement during group meetings, both in person and via videoconference. Our group of consumer representatives will contribute to dissemination of the trial findings.

### Ethics and dissemination

The PHOENIX trial is being undertaken in metropolitan Melbourne, Australia and a report of the trial findings will be prepared according to the consort Consolidated Standards of Reporting Trials statement for non-pharmacological interventions [64]. Our findings will be disseminated via presentations at scientific meetings and journal publications. The study is sponsored by The University of Melbourne, Australia and is coordinated and managed by staff based at Centre for Health, Exercise and Sports Medicine, Department of Physiotherapy. The sponsor has no role in the design of the trial. Any further modifications to the protocol, which may impact the study design and conduct, potential benefit or harm of the participants, will require a formal amendment to the ethics committee and the Australian New Zealand Clinical Trials Registry prior to implementation. Any additional modifications will be transparently reported or described when the findings of the trial are published.

### Trial status

Notification of funding was received in November 2018 and funding commenced in May 2019. The Human Research Ethics Committee of the University of Melbourne provided ethics approval in June 2019. Recruitment commenced in August 2019 and is anticipated to be complete in November 2022. The trial is due for completion in September 2023 when all participants are anticipated to have completed 9-month follow-up. Adjustments have been made to the study protocol predominantly due to COVID-19 and slow recruitment rates (see Additional File 3). Due to ongoing restrictions with COVID-19, we are unable to acquire all objective outcome measures but have proceeded with continuing the trial capturing self-reported outcome measures.

## Discussion

This protocol outlines the theoretical foundation and procedures for a two-arm superiority RCT comparing a combination of aerobic physical activity and strengthening exercise compared to strengthening exercise alone. There is biological plausibility that strengthening alone has limited potential to reduce hip OA symptoms for many people, and that adding aerobic exercise to strengthening exercise will result in better symptom relief with exercise. In a survey of 364 physiotherapists based in Australia, almost 95% frequently prescribed muscle strengthening exercise. In contrast, less than 45% of surveyed physiotherapists frequently prescribed aerobic activity for hip OA symptoms [65]. Clinicians may be more likely to prescribe aerobic exercise to manage people with hip OA if high-quality, direct evidence support a superior effect of a combination of aerobic and strengthening exercise compared to strengthening exercise alone.

Limitations of our trial will include our inability to i) determine if one type of aerobic physical activity is more beneficial than another and ii) determine if any potential between-group differences are attributable to aerobic physical activity per se and not simply more time spent being physically active. Nevertheless, this is the first clinical trial to determine if, and explore how, a combination of strengthening exercise and aerobic physical activity is more effective for hip OA symptom reduction than strengthening exercise alone.

## Supplementary Information

**Additional file 1.** Spirit Checklist.

**Additional file 2.** PHOENIX Protocol.

**Additional file 3.** PHOENIX Protocol Amendments.

**Additional file 4.** PHOENIX Consent.

**Additional file 5.** PHOENIX Strengthening Exercises.

## Data Availability

The datasets used and/or analyses during the current study will be available from the corresponding author (halm@unimelb.edu.au) on reasonable request once the study is completed.

## Abbreviations

ACSM: American College of Sports Medicine
AQoL: Assessment of Quality of Life
CHESM: Centre for Health, Exercise and Sports Medicine
COVID-19: Coronavirus disease of 2019
DASS: Depression Anxiety and Stress Subscale
MAF: Multi-Dimensional Assessment of Fatigue
MCID: Minimal Clinically Important Difference
NHMRC: National Health and Medical Research Council
NRS: Numeric Rating Scale
OA: Osteoarthritis
PSQI: Pittsburgh Sleep Quality Index
RCT: Randomised Controlled Trial
RPE: Rate of Perceived Exertion
WOMAC: Western Ontario and McMaster Universities Osteoarthritis Index
