# Supplementary material for: Effects of adding aerobic physical activity to strengthening exercise on hip osteoarthritis symptoms: protocol for the PHOENIX randomised controlled trial
Source: BMC Musculoskelet Disord. 2022 Apr 18;23:361. doi: 10.1186/s12891-022-05282-0 (PMC9014787; doi:10.1186/s12891-022-05282-0)
Supplement: Supplementary file 2 — Additional file 2. PHOENIX Protocol. [file 12891_2022_5282_MOESM2_ESM.pdf]

Combined strengthening and aerobic exercise versus strengthening for hip osteoarthritis symptoms: a randomised clinical trial

Authors
Dr Michelle Hall
Dr Kim Allison
Prof Kim Bennell
Prof Rana Hinman
Ms Gabrielle Knox
Ms Libby Spiers
Ms Fiona McManus
Dr Karen Lamb
A/Prof Ricardo Da Costa
Dr Melanie Plinsinga
Dr David Klyne
Dr Nick Murphy
Dr Fiona Dobson

Sponsor (TBC)
University of Melbourne

CONFIDENTIAL

This document is confidential and the property of the University of Melbourne.
No part of it may be transmitted, reproduced, published, or used without prior written authorisation from the institution.

STATEMENT OF COMPLIANCE

This document is a protocol for a clinical research study. The study will be conducted in compliance with all stipulations of this protocol, the conditions of ethics committee approval, the NHMRC National Statement on Ethical Conduct in Human Research (2007) and the Note for Guidance on Good Clinical Practice (CPMP/ICH-135/95).

Contents

STATEMENT OF COMPLIANCE 1
GLOSSARY OF ABBREVIATIONS 5
1. Study Management 5
2. INTRODUCTION AND BACKGROUND 9
2.1 Background Information 9
2.2 Research Question 10
2.3 Rationale for Current Study 10
3 STUDY OBJECTIVES 11
4. STUDY DESIGN 11
5. PARTICIPANT ENROLLMENT AND RANDOMISATION 20
5.1 Recruitment 20
5.2 Eligibility Criteria 20
5.2.1 Inclusion Criteria 20
5.2.2 Exclusion Criteria 21
5.3 Informed Consent Process 21
5.4 Enrolment and Randomisation Procedures 21
5.5 Blinding Arrangements 22
5.6 Participant Withdrawal 22
5.7 Trial Closure 22
5.8 Continuation of therapy 22
6. STUDY VISITS AND PROCEDURES SCHEDULE 23
7. CLINICAL AND LABORATORY ASSESSMENTS 24
8. INTERVENTION 25
9. ADVERSE EVENT REPORTING 26
9.1 Specific Safety Considerations (E.g. Radiation, Toxicity) 27
10. STATISTICAL METHODS 27
10.1 Sample Size Estimation 27
10.2 Population to be analysed 27
10.3 Statistical Analysis Plan 28
10.4 Interim Analyses 28
11. DATA MANAGEMENT 28
11.1 Data Collection & Storage 28
11.2 Data Confidentiality 29
11.3 Study Record Retention 29

12. ADMINISTRATIVE ASPECTS 29
12.1 Independent HREC approval 29
12.2 Participant reimbursement 29
12.3 Financial disclosure and conflicts of interest 30
13. USE OF DATA AND PUBLICATIONS POLICY 30
14. REFERENCES 31
15. APPENDICES 34

PROTOCOL SYNOPSIS

Title	**Combined strengthening and aerobic exercise versus strengthening for hip osteoarthritis symptoms: a randomised clinical trial**
Objectives	To compare the effects of a combined aerobic and muscle strengthening exercise program compared to a muscle strengthening exercise program alone on hip OA symptoms.<br><br>Primary: To determine whether a combination of aerobic and muscle strengthening exercise leads to significantly greater reductions in hip pain, and greater improvement in physical function compared to muscle strengthening only at 3 months.<br><br>Secondary: 1) To determine whether a combination of aerobic and muscle strengthening exercise leads to significantly greater reductions in hip pain, and greater improvement in physical function compared to muscle strengthening only at 9 months.<br><br>2) To determine whether a combination of aerobic and muscle strengthening has significantly greater benefits for other clinical outcomes (other measures of hip pain, cardiorespiratory fitness, body composition, health-related quality of life) compared to muscle strengthening only at 3 & 9 months.
Study Design	Comparative effectiveness participant- and assessor-blinded randomised controlled trial (RCT)
Planned Sample Size	196 participants
Selection Criteria	Participants aged 45 years and over with symptomatic hip OA
Study Procedures	Following baseline assessment, participants will be randomly allocated to receive either i) a 12-week combined aerobic and muscle strengthening exercise program or; ii) a 12-week muscle strengthening exercise program only.<br><br>Participants in both groups will be allocated to a physiotherapist for 9 online treatment sessions via videoconference over 3 months and will be asked to perform weekly exercise (3 strengthening exercise sessions per week at home) and up to 150 minutes of moderate intensity exercise for those in the combined aerobic and strengthening exercise group. Participants will be re-assessed at 3 months (laboratory visits & questionnaires) and 9 months (questionnaires).
Statistical Procedures<br>Sample Size Calculation:<br>Analysis Plan:	**Sample Size Calculation:** We aim to detect the minimal clinical important difference on the primary outcomes between groups (1.8 out of 10 units on numeric rating scale for overall pain and change in physical function of 6 units out of 68). We assume a between-participant standard deviation of 2.2 for pain and 13 units for physical function and a baseline to follow-up correlation of 0.46 for pain and 0.40 for physical function. To achieve 90% power and 5% significance level we require 83 participants per group. Allowing for a 15% loss to follow-up rate, we aim to randomise a total of 196 participants.

	**Analysis Plan:** A biostatistician will analyse blinded data. Main comparative analyses between groups will be performed using intention-to-treat. If there is >5% missing data, multiple imputation will be used to account for missing data. For the primary hypothesis, difference in mean change in pain and function (baseline minus follow-up) will be compared between groups using linear regression models adjusting for baseline values, with random effects to account for clustering by therapist. Similar analyses will be conducted for continuous secondary outcomes. Improvement based on global change will be compared across groups using risk differences, calculated from fitted logistic regression models. A sensitivity analysis will estimate treatment effects assuming full adherence, using a two-stage least squares approach. Effects of patient characteristics on outcomes will be explored by including relevant terms in models. Standard diagnostic plots will be used to check model assumptions. Effect modification by pre-specified baseline characteristics will be assessed through the inclusion of appropriate interaction terms in the regression models for the primary outcome. Mechanisms in accordance to pre-specified variables will be assessed using causal mediation analysis.
Duration of the study	Each participant will be involved for 9 months.

GLOSSARY OF ABBREVIATIONS

ABBREVIATION	TERM
AE	Adverse Events
ANCOVA	Analysis of Covariance
AQoL-6d	Assessment of Quality of Life instrument – 6-dimension
ACR	American Council of Rheumatology
ACSM	American College of Sports Medicine
BASE	Be Active Sleep Eat Facility, Monash University
BMI	Body mass index
CHESM	Centre for Health, Exercise and Sports Medicine
CPM	Conditioned Pain Modulation
DEXA	Dual-energy x-ray absorptiometry
KL	Kellgren & Lawrence (radiographic grading system)
HOOS	Hip Injury and Osteoarthritis Outcome Score
MCID	Minimal Clinically Important Difference
NHMRC	National Health and Medical Research Council
NICE	National Institute of Health and Care Excellence
NRS	Numeric Rating Scale
OA	Osteoarthritis
PASE	Physical Activity Scale for the Elderly questionnaire
PLS	Plain Language Statement
RCT	Randomized Controlled Trial
SD	Standard Deviation
RPE	Rate of Perceived Exhaustion

1. Study Management

1.1 Principal Investigator

Name	Role	Contact information
Dr Michelle Hall	Dr Hall is an exercise scientist with experience in osteoarthritis research. She will lead the RCT, co-supervise the Trial Coordinator, manage budget and be responsible for outcomes. She will be responsible for publishing the trial protocol and the findings of the trial.	P: 03 8344 40556<br>E: halm@unimelb.edu.au<br><br>Centre for Health Exercise and Sports Medicine<br>Department of Physiotherapy<br>School of Health Sciences<br>University of Melbourne VIC 3010

1.2 Associate Investigators

Name	Role	Contact information
Dr Fiona Dobson	Dr Dobson is a physiotherapist researcher who brings expert knowledge and skills in the area of osteoarthritis, outcome measures and management. She will co-supervise the Trial Coordinator.	P: 03 8344 8802<br>E: fdobson@unimelb.edu.au<br><br>Centre for Health Exercise and Sports Medicine<br>Department of Physiotherapy<br>School of Health Sciences<br>University of Melbourne VIC 3010
Ms Kim Allison	Dr Allison is a physiotherapist researcher who maintains an active clinical practice and brings specialised expertise in physiotherapy practice in hip osteoarthritis. She will oversee intervention according to predefined protocol and physical screening.	P: 03 8344 4860<br>E: kim.allison@unimelb.edu.au<br><br>Centre for Health Exercise and Sports Medicine<br>Department of Physiotherapy<br>School of Health Sciences<br>University of Melbourne VIC 3010
Prof Kim Bennell	Prof Kim Bennell is a research physiotherapist and Director of the Centre for Health, Exercise and Sports Medicine (CHESM). She will assist with developing the protocol, assist with trouble-shooting & interpretation of trial findings.	P: 03 8344 4135<br>E: k.bennell@unimelb.edu.au<br><br>Centre for Health, Exercise and Sports Medicine<br>Department of Physiotherapy<br>School of Health Sciences<br>University of Melbourne VIC 3010
Prof Rana Hinman	Prof Rana Hinman is a research physiotherapist and Deputy Director of the Centre for Health, Exercise and Sports Medicine (CHESM). She will assist with developing the protocol, assist with trouble-shooting & interpretation of trial findings.	P: 03 8344 3223<br>E: ranash@unimelb.edu.au<br><br>Centre for Health Exercise and Sports Medicine<br>Department of Physiotherapy<br>School of Health Sciences<br>University of Melbourne VIC 3010
Ms Gabrielle Knox	Ms Knox is a Research Scientist, who will take on the role of Trial Coordinator. This will include participant recruitment, administration of patient-reported, database management and scheduling of participant appointments.	P: 03 9035 4138<br>E: gabby.knox@unimelb.edu.au<br><br>Centre for Health Exercise and Sports Medicine<br>Department of Physiotherapy

		School of Health Sciences<br>University of Melbourne VIC 3010
Ms Libby Spiers	Ms Spiers is a research physiotherapist, who will assist with the drafting of study documents and manuals, ethics application and training of new staff for this study.	P: 03 9035 3886<br>E: libby.spiers@unimelb.edu.au<br><br>Centre for Health Exercise and Sports Medicine<br>Department of Physiotherapy<br>School of Health Sciences<br>University of Melbourne VIC 3010
Dr Melanie Plinsinga	Dr Plinsinga is an exercise scientist that is specialised in persistent, musculoskeletal pain, particularly tendinopathy and osteoarthritis. Dr Plinsinga is involved as a pain specialist in the trial.	P: 07 3735 8082<br>E: m.plinsinga@griffith.edu.au<br><br>Menzies Health Institute Queensland<br>Griffith University, QLD 4111
Dr Nick Murphy	Dr Murphy is an orthopaedic registrar at Central Coast Local Health District in NSW. He brings expertise in imaging and will grade the hip x-rays.	P: 04 0020 5454<br>E: nmur6094@uni.sydney.edu.au<br><br>Institute of Bone and Joint Research, Kolling Institute, University of Sydney.
Ms Fiona McManus	Ms McManus is a biostatistician. She will oversee all statistical analyses related to the trial and will assist with writing up of the trial findings.	E: fmcmanus@unimelb.edu.au<br><br>Centre for Epidemiology and Biostatistics<br>Melbourne School of Population and Global Health<br>Level 3, 207 Bouverie Street<br>Melbourne VIC 3010
Dr Karen Lamb	Dr Lamb is a biostatistician and senior research fellow. She will oversee all statistical analyses related to the trial and will assist with writing up of the trial findings.	E: klamb@unimelb.edu.au<br><br>Centre for Epidemiology and Biostatistics<br>Melbourne School of Population and Global Health<br><br>Methods and Implementation Support for Clinical Health Research Platform<br>Faculty of Medicine, Dentistry and Health Sciences<br>Level 3, 207 Bouverie Street<br>Melbourne VIC 3000
Dr David Klyne	Dr David Klyne is an expert in chronic pain and neuroimmunology. He will contribute to the selection of biomarkers for analysis, methods for analysis and interpretation.	P: 07 3365 4569<br>E: d.klyne@uq.edu.au<br><br>School of Health and Rehabilitation Sciences<br>University of Queensland, QLD 4067
A/Prof Ricardo Da Costa	A/Prof Ricardo Da Costa is an Associate Professor in Sports Dietetics and extremes physiology. A/Prof Da Costa will contribute to the collection and analysis of dual-energy	P: 03 9905 6861<br>E: Ricardo.costa@monash.edu<br><br>Department of Nutrition, Dietetics and Food

	x-ray absorptiometry (DEXA) data and cardiorespiratory fitness data.	Be Active Sleep Eat (BASE) Facility Monash University, VIC 3168

1.3 Statistician

Ms Fiona McManus	E: fmcmanus@unimelb.edu.au<br><br>Centre for Epidemiology and Biostatistics<br>Melbourne School of Population and Global Health<br>Level 3, 207 Bouverie Street<br>Melbourne VIC 3010
Dr Karen Lamb	E: klamb@unimelb.edu.au<br><br>Centre for Epidemiology and Biostatistics<br>Melbourne School of Population and Global Health<br><br>Methods and Implementation Support for Clinical Health Research Platform<br>Faculty of Medicine, Dentistry and Health Sciences<br><br>Level 3, 207 Bouverie Street<br>Melbourne VIC 3000

1.4 Internal Trial Committees

The Principal Investigator, the Trial Coordinator and at least one other Associate Investigator will meet fortnightly to monitor progress of the trial (Trial Management Committee), including any participant reports of adverse events.

1.5 Sponsor

The University of Melbourne.

1.6 Funding and resources

National Health and Medical Research Council (NHMRC) Project Grant #1159045 and University of Melbourne School of Health Science Mid-Career Researcher Grant (2019).

2. INTRODUCTION AND BACKGROUND

2.1 Background Information

Hip osteoarthritis (OA) affects one in four adults over their lifetime.[1] Pain and physical dysfunction are dominant, and often become more persistent and limiting as OA progresses. Hip OA substantially impairs quality of life. The 2016 Global Burden of Disease Study reported that OA is the 11th leading cause of disability worldwide.[2] Osteoarthritis affects around 2 million Australians, with estimates predicting this will rise to 3.1 million Australians by 2030.[3] Healthcare system costs related to arthritis exceeded $2.1AUD billon in 2015 and are forecast to reach $2.9 billion by 2030[4] of which OA is the largest contributor. The greatest driver of health care cost for hip OA is joint replacement surgery. The societal and healthcare burden of OA will continue to rise due to the ageing population and escalation in obesity rates.[2] The Australian Government has designated arthritis a National Health Priority and is developing a National Action Plan to reduce disease burden.

There is no cure for OA. Non-drug and non-surgical treatment are central to managing hip OA. Exercise is endorsed by all OA clinical guidelines as central to self-management.[5] However, the effect

of exercise on hip OA symptoms is small-to-modest and does not provide clinically meaningful improvements for many patients.[6] Evidence to date is largely based upon lower-limb muscle strengthening exercise. However, muscle strengthening alone has limited potential to improve hip OA symptoms.[7] Poor cardiovascular health and psychological well-being are often reported in people with hip OA and are associated with greater severity of hip OA symptoms.[8, 9] Both cardiovascular health and psychological well-being can be improved to a greater extent with aerobic exercise compared to muscle strengthening exercise.[10, 11] Therefore, the addition of aerobic exercise (e.g. walking, swimming, cycling) to lower-limb muscle strengthening exercise may have a greater benefit on hip OA symptoms compared to lower-limb muscle strengthening exercise alone. There are no head-to-head RCTs substantiating the superior effect of combined exercise types compared to lower-limb muscle strengthening.

2.2 Research Question

Is there a difference in clinical outcomes at 3 and 9 months between an exercise program combining aerobic and strengthening exercise, compared to strengthening only, in people with painful hip OA?

2.3 Rationale for Current Study

Exercise is advised for all people with hip OA irrespective of age, OA severity, pain, function and co-morbidities.[5] A 2017 meta-analysis of exercise RCTs in hip OA identified 12 RCTs and showed small-to-modest beneficial effect of exercise on pain (effect size -0.24, 95%CI: -0.42, -0.06) and physical function (effect size -0.34, 95% CI: -0.50, -0.18) compared to no exercise.[6] Importantly, the effect of exercise does not reach minimal clinically important improvement in symptoms. Of note, all existing RCT exercise trials include lower-limb strengthening, while few trials (k=3) include aerobic exercise. Thus, current exercise evidence is predominately based on lower-limb strengthening interventions, which is highly likely to account for the reported small-to-modest beneficial effects of exercise on hip OA symptoms.

Hip and thigh muscle weakness is widely established in people with hip OA.[12] However, muscle strengthening exercise in isolation is likely inadequate to fully alleviate pain and physical dysfunction associated with hip OA. Supporting this, our team has investigated relationships between lower-limb muscle strength and hip OA symptoms.[7, 13] In a cohort of 195 patients with hip OA, we found that very large and unfeasible increases in muscle strength are likely required to achieve clinically meaningful improvements in symptoms for many patients.[7] These findings highlight the limits of muscle strengthening exercise in isolation to improve hip OA symptoms.

Poor cardiovascular fitness and psychological well-being are associated with greater hip OA symptom severity.[8, 9] Cardiovascular health and psychological well-being can be improved to a greater extent with aerobic exercise compared to muscle strengthening exercise.[10, 11] Therefore, incorporating aerobic exercise into lower-limb muscle strengthening programs may yield better symptomatic benefits for hip OA compared to lower-limb strengthening alone. Clinical trials have demonstrated the superior effect of aerobic exercise on cardiovascular fitness compared to strengthening exercise[14], including in hip OA.[10] Of particular relevance is an RCT of 141 obese adults without OA, which demonstrated that a combination of aerobic and strengthening exercise improved cardiovascular fitness to a greater extent than strengthening exercise alone.[14] This is important because improved cardiovascular fitness is significantly associated with improved walking speed[15],a key determinant of physical function for people with hip OA.[16]

Aerobic exercise also leads to greater improvement in psychological health compared to strengthening exercise alone. In hip OA, a recent RCT including 162 participants found greater improvements in overall mental health and self-efficacy (belief in ability) with aerobic exercise, as well as greater improvements in physical function, compared with strengthening exercise.[10]

Depressed mood leads to increased disability over time among people with hip OA.[17] Despite a paucity of investigations in hip OA, high-quality evidence supports the positive effects of aerobic exercise on mood.[18] An RCT in knee OA established that aerobic exercise had greater benefits on mood compared to strengthening exercise.[11] The benefit of aerobic exercise on mood enhancement, was observed irrespective of baseline mood status. Collectively, the evidence strongly supports the hypothesis that adding aerobic exercise to a lower-limb strengthening program will optimise clinical outcomes for people with hip OA. However, there are no RCTs evaluating the additive benefits of aerobic exercise to a lower-limb muscle strengthening program, compared to a lower-limb muscle strengthening program alone.

3 STUDY OBJECTIVES

3.1 Primary Objective

To determine if a combination of aerobic and lower limb muscle strengthening exercise will lead to a greater reduction in hip pain and greater improvement in physical function at 3 months compared to lower limb muscle strengthening exercise alone.

3.2 Secondary Objectives

To determine if a combined program of aerobic and muscle strengthening exercise will lead to a greater reduction in hip pain and greater improvement in physical function at 9 months compared to a muscle strengthening exercise program alone.

To determine if a combined program of aerobic and muscle strengthening exercise will have greater benefits for other clinical (e.g. quality of life), physical function (e.g. 40-meter fast pace walk test), and psychological outcomes (e.g. mood) at 3 & 9 months compared to a muscle strengthening exercise program alone.

4. STUDY DESIGN

4.1 Type of Study

Comparative effectiveness participant- and assessor-blinded randomised controlled trial (RCT).

4.2 Study Design

This two-arm RCT will be conducted at The University of Melbourne. A total of 196 participants with clinically-diagnosed hip OA (see selection criteria below) will be recruited from the community via advertisements, print/radio/social media, clinicians and our volunteer database. Participants will be randomly allocated to either a i) 12-week exercise program combining strengthening and aerobic exercise; ii) a 12-week strengthening exercise program.

4.3 Number of Participants

We will recruit 98 participants per treatment arm, therefore 196 participants will be recruited in total.

4.4 Study sites

The study will be conducted at The University of Melbourne. The physiotherapy consultations will be delivered online via videoconference (Zoom) by private practitioners across Melbourne.

4.5 Expected Duration of Study

Anticipated start date for recruitment: July 2019
Anticipated end date for data collection: Dec 2023

We anticipate recruitment for this study will take approximately 36 months (approx. 6 participants per month, based on data from the HOPE trial & our pilot study previously completed in CHESM). Thus, we expect recruitment to take place between July 2019 to June 2022.

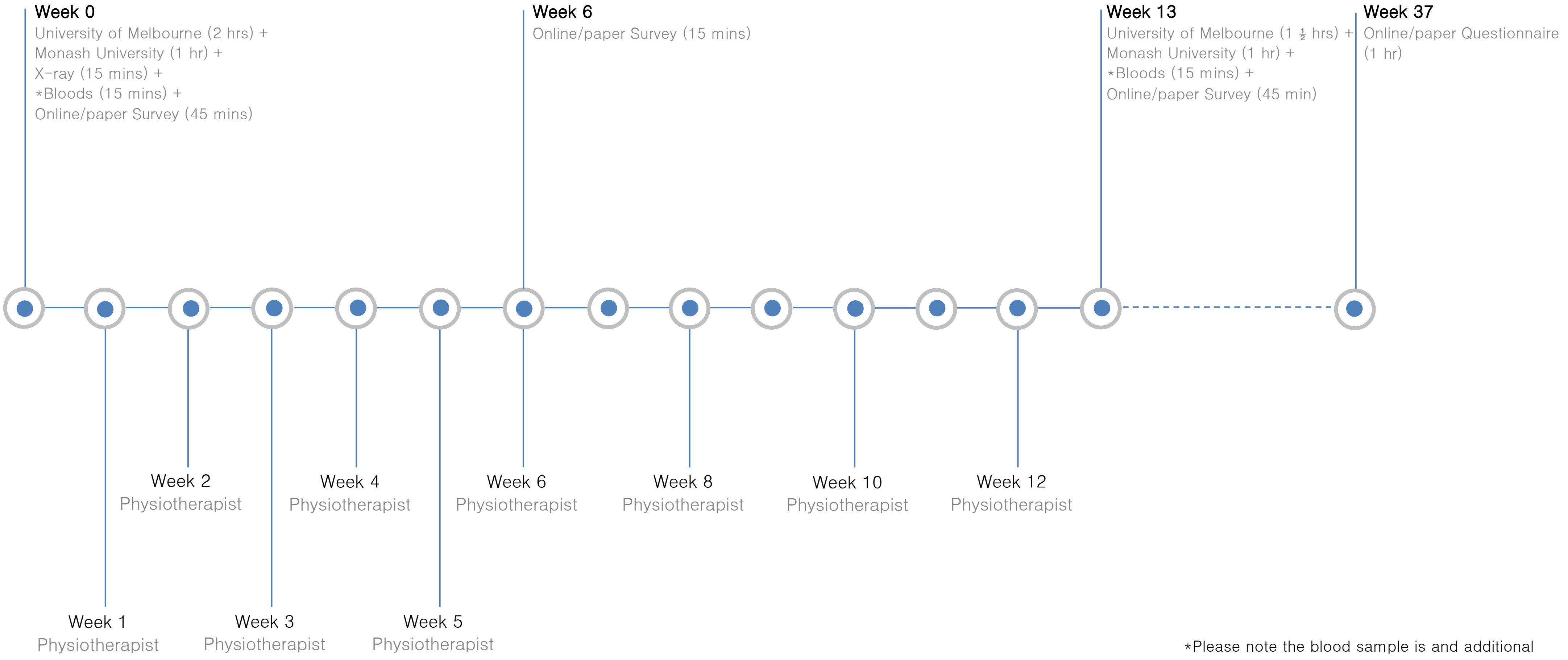

Figure 1. Overview of appointments

*Please note the blood sample is and additional measure that participants can opt-in to take part.

+These appointments will only take place if permitted and deemed safe by the University and the Victorian Government (COVID-19)

4.6 Primary and Secondary Outcome Measures

Name	Description	Scale	Time-points measured
**Primary Outcomes**			
Severity of overall hip pain[19]	Scored on an 11-point numerical rating scale (NRS) for average overall hip pain in the last week.	Ranges from 0 to 10; where 0=no pain and 10=worst pain possible.	Baseline, 3 months and 9 months
Physical function subscale of the Western Ontario & McMaster Universities Osteoarthritis Index (WOMAC)[20]	Scored using 17 questions regarding hip function in the last 48 hours with Likert response options ranging from None to Extreme.	Total score ranges from 0 to 68; higher scores indicate worse function.	Baseline, 3 months and 9 months
**Secondary Outcomes**			
Global improvement in:<br>a) Hip pain,<br>b) Physical function	Scored using a 7-point global rating of change Likert scale from with response options ranging from “much worse” to “much better” when compared to baseline.	Participants who indicate that they are “moderately better” or “much better” will be classified as improved. All other respondents will be classified as not improved.	3 months and 9 months
Patient specific functional scale	Up to five activities of daily living are identified and scored on an 11-point NRS to record if performance was limited in the previous week.	Ranges from 0 to 10; where 0=unable to perform and 10=able to perform the activity at the same level as before hip osteoarthritis. Higher scores indicate better function.	Baseline, 3 months and 9 months
Quality of life (AQoL-6D)[21]	Scored using the 20-item Assessment of Quality of Life II Instrument (6D version), which covers the topics of Independent Living, Relationships, Mental Health, Coping, Pain and Senses to come up with one overall value representing quality of life.	Total score ranges from −0.04 to 1.00; higher scores indicate better quality of life.	Baseline, 3 months and 9 months
Self-efficacy for walking[22, 23]	Perceived ability to walk at various durations and overcoming obstacles (e.g. stairs) will be measured by the Self-Efficacy for Walking Scale-Duration and the modified Gait Self-Efficacy Scale.	Total score for each measure of self-efficacy is then calculated by summing the confidence rating and dividing by total number of items in the Scale, resulting in a maximum possible efficacy score of 100.	Baseline, 3 months and 9 months
Brief Fear of Movement Scale for Osteoarthritis[24]	Scored from a 6-question self-reported disease-specific instrument to assess activity avoidance due to pain related fear of movement	6 items scored on a 4-point scale from ‘strongly disagree’ to ‘strongly agree’. Higher scores indicate higher few of movement	Baseline, 3 months and 9 months
DASS-21 Depression Subscale[25]	Scored from a 7-item scale. Rated on a 4-point Likert scale from 0= ‘did not apply	Range 0-42 with higher scores indicating higher levels.	Baseline, 3 months and 9 months

	to me at all' to 3 = 'applied to me very much or most of the time'.		
DASS-21 Anxiety Subscale[25]	Scored from a 7-item scale. Rated on a 4-point Likert scale from 0= 'did not apply to me at all' to 3 = 'applied to me very much or most of the time'.	Range 0-42 with higher scores indicating higher levels.	Baseline, 3 months and 9 months
Pittsburgh Sleep Quality Index (PSQI)[26]	Scored from a 7-component self-reported instrument which measure the quality and patterns of sleep. Rated on a 3-point scale, whereby 3 reflects the negative extreme on the Likert Scale.	Range form 0-27 with a global sum of 5 or greater indicates a poor sleeper.	Baseline, 3 months and 9 months
Multi-Dimensional Assessment of Fatigue (MAF)[27]	Scored from a 16-item scale that cover four dimensions of fatigue (degree and severity, distress, frequency and change over the past week, and its impact on daily living)	Items 1-15 are used to calculate the global fatigue index (GFI). Range 1-50 with higher scores indicating more severe fatigue.	Baseline, 3 months and 9 months
*Hip extensor strength[28]	Maximum voluntary isometric strength will be assessed using hand held dynamometer (Lafayette Manual Muscle Test System) with participants in supine and hip in a neutral position. Mean torque over 2 maximal efforts lasting 3 seconds will be recorded (Nm/kg)	Higher score indicates greater strength.	Baseline and 3 months
*Knee extensor strength [29]	Maximum voluntary isometric strength will be assessed using an isokinetic dynamometer with the knee at 60 degree knee flexion. Peak torque over 3 maximal efforts lasting 5 seconds will be recorded (Nm/kg)	Higher score indicates greater strength.	Baseline and 3 months
*Hip abductor strength [28]	Maximum voluntary isometric strength will be assessed using hand held dynamometer (Lafayette Manual Muscle Test System) with participants in supine and hip in a neutral position. Mean torque over 2 maximal efforts lasting 3 seconds will be recorded (Nm/kg)	Higher score indicates greater strength.	Baseline and 3 months
*30 second chair sit to stand test[13]	Number of complete chair stands completed in 30 secs	Higher score indicates better function.	Baseline and 3 months
*40 meter fast pace walk test[13]	Time to taken to walk 4 x 10 m quickly but safely (m/s)	Higher speed indicates better function.	Baseline and 3 months
*6-step stair climb test[13]	Time take to ascend and descend a flight of 6 stairs as	Shorter time indicates better function.	Baseline and 3 months

	quickly as possible. Use of handrail is permitted if needed.		
*Cardiorespiratory fitness[30]	Prior to this assessment hydration is assessed using a bioelectrical impedance scan. However, if participants have a non-titanium metal implant they will be asked to provide a urine sample prior to cardiorespiratory assessment instead. Cardiorespiratory fitness will be assessed as submaximal oxygen consumption using a graded incremental exercise protocol using a cycle ergometer. Participants will start at 1W/kg and increase 0.5W/body mass (kg). Test will terminate when respiratory quotient is >1.00, or equal to ~75% of max heart rate.	Oxygen consumption in ml per min relative to body mass will reported. Higher values indicate greater cardiorespiratory fitness.	Baseline and 3 months
*Body composition	Total body mass, fat mass and lean mass will be measured using dual-energy x-ray absorptiometry (DEXA). Participants will be asked to fast 2 hours before the scan.	Absolute values will be reported in kg. Higher score indication more fat mass and more lean mass	Baseline and 3 months
Adherence to consultation with physiotherapist	Recorded by physiotherapist for each consultation	Number of consultations attended (face-to-face or telephone)	Throughout the initial 3 months
Adherence with strengthening exercise program	Obtained from participant log book (Range from 0 to 36 session). Reported in whole numbers.	Higher indicates more strengthening exercise completed	Throughout the initial 3 months
Number of minutes during at least moderate intensity exercise	Participants will be instructed to wear a wearable physical activity monitor over the initial 3 months of the study period. Number of minutes during at least moderate intensity exercise will be based on heart rate data. With participant consent, de-identified daily heart rate data will be automatically uploaded to a cloud-based system (Fitabase – Small Step Lab) from the participant's wearable activity monitor account.	Higher indicates greater time being active	Throughout the initial 3 months
Co-intervention use	Participants will complete a custom-developed table to	Participants who indicate they have used a	Baseline, 3 months and 9 months

	indicate the frequency of use (over the past 6 months) of a range of pain and arthritis medications and co-interventions.	drug/supplement at least once a week in the past month will be reported as a current user of the relevant medication. Participants who have used a co-intervention once in the past 6 months will be reported as a current user.	
Adverse events	Recorded by participants using a custom-developed table.	Proportions of participants experiencing adverse events will be reported.	3 months and 9 months
Expectation of treatment outcome	Rated using a Likert scale with anchors of "no effect at all" to "complete recovery"	The number and proportion of participants selecting each response option will be reported.	Baseline
Overall satisfaction with the exercise program	Scored on a Likert Scale for "Overall how satisfied are you with the program you received for your hip pain? From 0 = 'not at all satisfied' to 10 = 'extremely satisfied'.	Higher indicates greater satisfaction	6 weeks and 3 months
Working Alliance Inventory Short Form[31]	Scored separately by the participant and physiotherapist after 6th consultation, based on 12 statements relating to the perceived trust and agreement between the therapist and client, using a 7-point scale.	Overall scores range from 12 to 84 (with higher scores indicating a stronger therapeutic alliance)	6 weeks
Overall hip pain during walking	Scored on an 11-point numerical rating scale (NRS) for overall hip pain during walking in the last week.	Ranges from 0 to 10; where 0=no pain and 10=worst pain possible.	Baseline, 3 months and 9 months
Western Ontario & McMaster Universities Osteoarthritis Index (WOMAC) Pain subscale[20]	Scored using 5 question regarding hip pain in the last 48 hours with Likert response options ranging from None to Extreme.	Ranges from 0 to 20; higher scores indicate worse pain	Baseline, 3 months and 9 months
Western Ontario & McMaster Universities Osteoarthritis Index (WOMAC) Stiffness subscale[20]	Scored using 2 question regarding hip stiffness in the last 48 hours with Likert response options ranging from None to Extreme.	Ranges from 0 to 8; higher scores indicate worse stiffness	Baseline, 3 months and 9 months
DASS-21 Stress Subscale[25]	Scored from a 7-item scale. Rated on a 4-point Likert scale from 0= 'did not apply to me at all' to 3 = 'applied to me very much or most of the time'.	Range 0-42 with higher scores indicating higher levels.	Baseline, 3 months and 9 months
Arthritis Self Efficacy Scale[32]	Participants rate their ability to do 8 tasks from 1 (very uncertain) to 10 (very certain).	Total scores are an average of the 8 items with a range from 1 to 10; higher scores indicate higher self-efficacy.	Baseline, 3 months and 9 months

Motivation for Exercise Self-Report Behavioural Automaticity Index[33]	Scored from a 4-item self-reported instrument. Rated on a 7-point scale from 1 = 'strongly disagree' to 7 = 'strongly agree'.	Higher score indicates higher levels of behavioural automaticity	Baseline, 6 weeks, 3 months and 9 months
Motivation for Exercise Intention Decision and Strength[34]	Scored from a 2-item self-reported instrument. Rated on a 7-point scale from 1 = 'not at all' to 7 = 'very much'.	Higher score indicates stronger intention to exercise	Baseline, 6 weeks, 3 months and 9 months
Motivation for Exercise Instrumental and Affective	Scored from a 2-item self-reported instrument. Item one will be rated on a 7-point scale from 1 = 'harmful' to 7 = 'beneficial'. Item two will be rated on a 7-point scale from 1 = 'not enjoyable' to 7 = 'enjoyable'.	Higher score indicates higher levels of affective judgement to exercise	Baseline, 6 weeks, 3 months and 9 months
**Baseline measures**			
Co-morbid conditions	Self-administered comorbidity questionnaire[35]	The number and proportion of comorbidities will be reported.	Baseline
**Height	Measured in the laboratory using a stadiometer	Measured in metres	Baseline
**Weight	Measured in the laboratory using scales	Measured in kilograms	Baseline
Body mass index (BMI)	Calculated from height and weight.	Reported in kg/m^2	Baseline
Age	Calculated from date of birth.	Reported in years	Baseline
Gender	Self-report of identified gender	Male or female or not disclosed	Baseline
Unilateral hip symptoms	Participant will self-report if their symptoms are in one or both hips.	Reported as number and percentage	Baseline
Duration of hip symptoms in the study hip	Participants will self-report the total duration of time since their study hip symptoms began.	Reported in years	Baseline
*Radiographic disease severity of hip osteoarthritis	Rated from a non-weight bearing x-ray using the KL scale[36]	The number and proportion of participants with Grade 2 (mild disease severity), 3 (moderate disease) and Grade 4 (severe disease) will be reported.	Baseline
Current employment status	Participants will report their current employment status using a categorical scale with response option currently employed; retired (not due to health reasons); unemployed/student; homemaker; unable to work due to health reasons.	The number and proportion of respondents for each category will be reported.	Baseline
Education level	Participants will report their current education level using	The number and proportion of respondents	Baseline

	a categorical scale with response options < 3 years of high school; 3 or more years of high school; some education beyond high school; completed tertiary or higher education	for each category will be reported.	
A range of additional measures will be collected for the purposes of answering questions about pain, and for subsequent analyses of potential mediating effect on clinical outcomes. These measures will not be used, however, to determine treatment efficacy.			
*Quantitative Sensory Assessment: Pressure pain threshold[37]	Pain pressure threshold (PPT) will be assessed using pressure algometer with probe size 1 cm^2 and rate of pressure increase of 30 Pa/s. The probe is pressed into the greater trochanter of the femur with gradual increase of pressure. The test stops once the sensation of pressure changes to pain (i.e. threshold). 3 reps with 30 sec interval and mean value will be calculated.	Higher value indicates less sensitivity to pain.	Baseline and 3 months
*Quantitative Sensory Assessment: Wind up Ratio	The perceived intensity of a single pinprick stimulus (256 mN pinprick) will be compared to a series of 10 repetitive stimuli of the same intensity, applied at a rate of 1/sec with and area of 1 cm^2. Participants will be asked to give pain rating for the single stimulus and a pain rating for the series of 10 stimuli. Repeated 5 times. Mean value will be calculated	Higher values indicate greater sensitivity to pain.	Baseline and 3 months
*Quantitative Sensory Assessment: Conditioned pain modulation (CPM)[37]	CPM will be assessed as the change in pain perceived in one body region as a result of pain induced in another body region.PPT will be measured over the test hip. Participants will immerse their contralateral foot of the measured side in cold water at ~12 degrees. When patient reports a pain intensity between 4-6 out of 10 the hand is removed from cold water and PPT will be measured again, the difference between pre and post measures the CPM.	Higher values indicates greater abnormality in pain modulation.	Baseline and 3 months
*Inflammatory cytokines	Blood samples will be acquired and analysed for IL-6, TNF-alpha, CRP and other	Higher values indicate higher inflammation.	Baseline and 3 months

	inflammatory markers to be determined. Participants will be asked to fast 12 hours before providing blood sample.		
Spreading of pain[38, 39]	Participants will be asked to shade body sites with the pain and determine and answer 14 items about their pain.	Number of pain sites will be used to quantify the spreading of pain and question will determine likelihood of neuropathic pain.	Baseline, 3 months and 9 months
Please note:<br>*These secondary outcomes will only be collected if face-to-face assessments are permitted and deemed safe by the University of Melbourne and the Victorian Government (COVID-19).<br><br>**These secondary outcomes will be collected in the CHESM Human Movement Laboratory at the University of Melbourne if face-to-face assessments are permitted and deemed safe by the University of Melbourne and the Victorian Government (COVID-19). Alternatively, these secondary outcomes will be self-reported if face-to-face assessments are not permitted or deemed safe by the University of Melbourne and the Victorian Government.			

5. PARTICIPANT ENROLLMENT AND RANDOMISATION

5.1 Recruitment

Participants will be recruited from the community via advertisements, print/radio/social media, clinicians and our volunteer database. Volunteers will complete an online screening form, then those who are potentially eligible will undertake follow-up screening over the phone with the Trial Coordinator.

5.2 Eligibility Criteria

5.2.1 Inclusion Criteria

Participant will be eligible for the study if they meet the following inclusion criteria:

Participants will be eligible for the study if they meet the following inclusion criteria:

i. National Institute for Health and Care Excellence[40] clinical criteria for OA;
   a. Age ≥45 years;
   b. Activity-related hip joint pain;
   c. morning stiffness ≤30 minutes;

ii. report history of hip pain >3 months;
iii. report hip pain on most days of the past month;
iv. report an average overall pain score of at least 4 on an 11-point numeric rating scale (anchored at 0=no pain, 10=worst pain imaginable) over the previous week;
v. pass the American College of Sports Medicine Exercise Pre-participation Health Screening Questionnaire[41]; or obtain general practitioner clearance for participation in this study *see Phoenix Exercise Clearance GP letter*);
vi. access to a device with internet connection.

For participants with bilateral eligible hips, the most symptomatic hip will be deemed the study hip with respect to outcome assessment.

5.2.2 Exclusion Criteria

i) unable or unwilling to comply with study protocol;
ii) inability to speak English;
iii) on waiting list or planning back/lower limb surgery in the next 12 months;
iv) previous hip replacement in the affected hip;
v) any hip surgery in the past 6 months;
vi) currently taking corticosteroids or have done so in the past 3 months;
vii) any hip injections in the past 3 months or planned injections in next 9 months;
viii) participating in strengthening exercises at least 3 times per week and/or engaging in 150 minutes of moderate aerobic exercise per week within past 6 months;
ix) self-reported inflammatory arthritis;
x) any neurological condition affecting lower limb and ability to exercise safely;
xi) any unstable or uncontrolled cardiovascular condition
xii) pregnancy or planned pregnancy.

5.3 Informed Consent Process

The Chief Investigator will ensure that the Trial Coordinator is trained in obtaining informed consent for this research. All potential participants will receive verbal and written information about the purposes, potential risks and processes involved in the study from the Trial Coordinator. In accordance with the latest revision of the World Medical Association Declaration of Helsinki, informed consent will be obtained from all participants by signing a consent form after the researcher is certain that the participant understands the information delivered and has had an opportunity to ask questions and before proceeding with the baseline assessments.

Volunteers will initially be screened by an online form, then a subsequent telephone screening will involve a detailed verbal description of the project to ensure that participants are comfortable with and able to comply with trial procedures and have had the opportunity to have all questions answered. Participants deemed eligible at this stage will be sent the Plain Language Statement (PLS) and Consent Form in the post or by email (via REDCap).

Participants will be encouraged to phone researchers if they have any questions or concerns regarding the contents of the PLS and/or Consent Form. After reading the PLS, and if they give their consent to participate, they will sign the consent form online via REDCap or return it via a reply-paid envelope in the post or by scanning and emailing the document to the Trial Coordinator.

To ensure participant blinding (see below), the PLS and consent processes will involve limited disclosure about the specific exercise programs under investigation and study hypotheses. Participants will be informed that the trial is evaluating two different undisclosed “types” of exercise to compare effects on hip OA symptoms. Participants will not be told about the specific characteristics of the exercise under investigation.

5.4 Enrolment and Randomisation Procedures

Screening will be conducted via online screen then over the telephone as outlined above. Participants will be enrolled into the study once the informed consent process has been completed and they have completed the baseline questionnaire (on a web-based platform (REDCap) or via post) and baseline assessment at the University of Melbourne. If face-to-face appointments are not permitted by the University of Melbourne and the Victorian government (i.e. COVID-19), participants will be enrolled into the study once the baseline questionnaire has been completed. Each participant will receive a unique study ID and this will be documented in the participant’s record/database in addition to all study documents.

The randomisation schedule will be prepared by the biostatistician (permuted block sizes 6 to 12) stratified according to therapist (to control for therapist variation). The schedule will be stored on a password-protected website (REDCap) maintained by a researcher not involved in either participant recruitment or administration of primary/secondary outcome measures. Group allocation will be revealed by this same researcher after baseline primary/secondary outcomes have been completed.

5.5 Blinding Arrangements

Participants will be blinded to group allocation by the process of limited disclosure (see above). Participants will not be informed about the study hypotheses, or which group they were allocated to, until the study is completed, at which time they will be provided a lay summary of study purpose, hypotheses and findings. As the primary outcomes are participant-reported, and participants are blinded, the primary outcomes are considered assessor-blinded. Secondary outcomes consist of both participant-reported and objective measures which will also be considered outcome assessor blinded. Research staff entering the data will be blinded. Statistical analyses will be performed blinded. Descriptive baseline characteristics that are researcher-measured using objective methods in the laboratory (e.g. height, weight, physical function performance, muscle strength, cardiorespiratory fitness and body composition) will be measured by a blinded researcher. Physiotherapists will also be unblinded to the study hypotheses.

5.6 Participant Withdrawal

As participation in this study is voluntary, participants may withdraw from i) participating in either exercise program; and/or ii) continuing with scheduled data collection processes, at any point over their 9-month involvement. Participants may choose to stop attending physiotherapy sessions and/or exercising but continue with some/all aspects of data collection. To minimise data loss, research staff will encourage participants to complete primary outcome measures (at a minimum) at the 3-month time frame, over the telephone if necessary. If a participant withdraws from the study, the nature, timing of and reasons for withdrawal will be recorded (provided the participant responds to contact made by the research team). Any data provided up to the point of withdrawal will be kept in accordance with intention-to-treat analyses, unless the participant specifically requests to withdraw all of their data from the study.

5.7 Trial Closure

For each participant, the follow-up duration is 9 months from randomisation. At this point, final assessment will take place and no further data collection or monitoring will occur for that participant.

5.8 Continuation of therapy

As participants will keep their equipment (e.g. ankle cuff weight, resistance bands) after their involvement in the study has ended, they may choose whether they wish to continue exercising, or not.

6. STUDY VISITS AND PROCEDURES SCHEDULE

Study Flow Chart

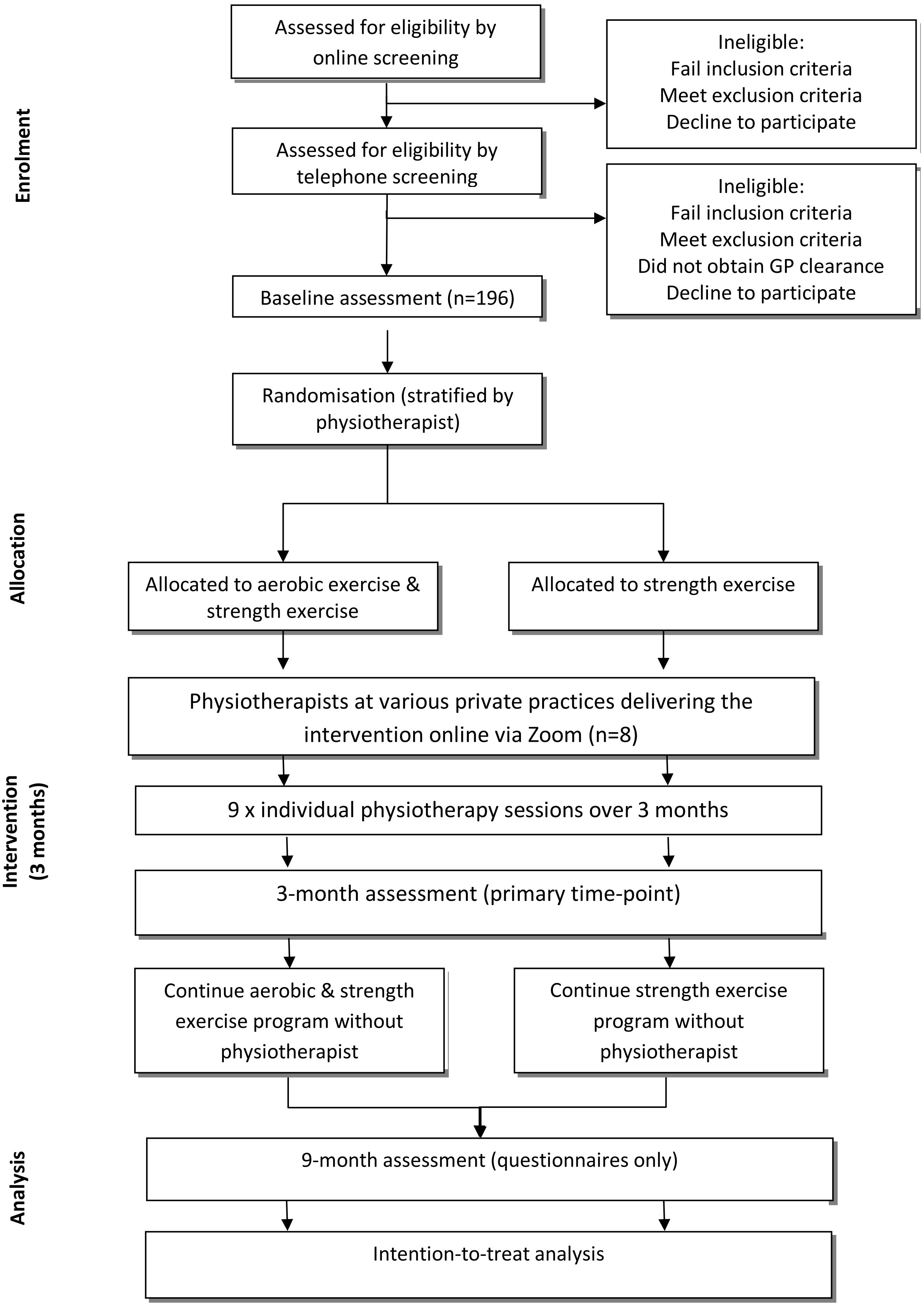

7. CLINICAL AND LABORATORY ASSESSMENTS

Figure 1 outlines clinical and laboratory assessment schedule. Only when permitted by the University of Melbourne (i.e. due to COVID-19) will any face-to-face assessments be scheduled for participants. Following enrolment the Trial Coordinator will i) schedule a testing appointment at the University of Melbourne; ii) schedule a cardiorespiratory fitness and body composition appointment at the Be Active Sleep and Eat (BASE) Facility at Monash University; iii) provide a referral and schedule a x-ray appointment [available sites include: Blackburn South Radiology; Bridge Road Imaging; Brunswick Diagnostic Radiology; Healthscan Specialist Imaging Narre Warren; Healthscan Specialist Imaging Ringwood], and; iv) provide a referral for blood collection [all 298 Melbourne Pathology Clinics are available to participants]. The Trial Coordinator will also schedule the first physiotherapy appointment via Zoom. The Trial Coordinator will provide materials (Participant Zoom Information Booklet) to prepare participants to use Zoom prior to their first consultation. At 3-months, participants will be asked to complete the questionnaire booklet online. If participants completed face-to-face assessment at baseline, and if face-to-face appointments are permitted, participants will also be asked to return to i) the University of Melbourne, ii) Monash University and ii) Melbourne Pathology at 3-months. At 9-months, participants will only be asked to complete questionnaires online or hard copy if they prefer. Please note that participants will be asked to opt-in for blood sample assessment at baseline and 3-month follow-up.

X-rays (see Section 4.6)

If a participant has not had an anteroposterior x-ray of their hip in the past 12 months, they will be asked to attend one of the participating radiology clinics in Melbourne to undergo a standardised anteroposterior (AP) supine hip x-ray. Participants who have undergone a weightbearing or supine anteroposterior or posteroanterior x-ray in the previous 12 months and can provide the images to research staff for screening, will not undergo new x-rays due to ethical concerns of exposing them to unnecessary radiation. Participants who have their own recent suitable hip x-ray will be provided a stamped return envelope to post the x-rays to the research team.

Patient-reported outcomes (see Section 4.6)

At baseline, participant-reported outcomes will be collected electronically in REDCap via computer or on paper via post (if preferred). At 3- and 9-month follow-up, participants will be sent questionnaires electronically (or on paper if preferred) to complete primary and secondary self-reported outcomes at home.

Cardiorespiratory fitness & body composition (see Section 4.6) (if permitted)

Participants will be asked to attend the Be Active Sleep Eat (BASE) Facility at Monash University to undergo submaximal cardiorespiratory fitness assessment and a DEXA scan to assess body composition assessment at baseline and 3-month follow-up.

University of Melbourne laboratory-based outcomes (see Section 4.6) (if permitted)

At baseline, laboratory-based secondary outcomes will be collected in the CHESM laboratory. At 3-month follow-up, participants will be asked to return to the CHESM laboratory to complete the secondary outcomes. Some of the measures outlined are opt-in.

Inflammatory outcomes (see Section 4.6) (if permitted)

At baseline, participants will be asked if they agree to opt-in to have a blood sample taken at any of the Melbourne Pathology centres. The blood will be stored at Melbourne Pathology and analysed for inflammatory markers. At 3-month follow-up participants will be asked to return to Melbourne Pathology to have another sample of blood taken.

8. INTERVENTION

This 9-month study includes 3-month exercise programs delivered by a physiotherapist followed by a continuation of the exercise program independently for a further 6 months. Participants in both groups will attend 9 online treatment sessions via Zoom, over the first 3 months. Once per week over the first 6 weeks and fortnightly thereafter. The first session will be up to one hour with subsequent sessions up to 30 minutes. Physiotherapists will provide participants with home-based strengthening exercises in paper format, containing exercise instructions. Participants in both groups will be prescribed exercise dosage (i.e. frequency, intensity, time) according to the American College of Sports Medicine (ACSM) recommendations. A 2017 meta-analysis in this area demonstrated that exercise programs prescribed in accordance with ACSM dosage recommendations resulted in greater symptom improvement compared to exercise programs with questionable concordance to ACSM dosage recommendations.[6] Feasibility and acceptability of aerobic and strengthening exercise prescribed according to ACSM dosage recommendations has been demonstrated in our recent pilot study (HREC. 1851762 Participants will be provided with an ankle-cuff weight and resistance bands. Participants will also receive a wearable activity monitor to wear daily for the initial 3 months of the study.

Strengthening only: Participants will be instructed by the physiotherapist how to perform 5-6 lower limb strengthening exercises, including how to use equipment such as ankle cuff weights and exercise resistance bands. Initial sessions will involve 1 to 2 sets of 8 to 12 repetitions at an intensity deemed by the participant to be at least 'hard' (=<7) on the modified Borg Scale of rating of perceived exertion (RPE). The dosage will increase as appropriate to 3 sets at 'hard to very hard' (between 7 to 8) on modified RPE Borg Scale (*Modified Borg Scale; 10 is maximal effort*). Strengthening exercises will take approximately 30 minutes per session. Participants will be asked to complete these three times per week at home.

Aerobic & strengthening: Participants will be prescribed muscle strengthening exercise as above. They will also be facilitated to build up to engaging in 150 minutes of moderate intensity aerobic exercise per week, which will be tailored to individual preferences, including a choice of activities such as walking, cycling and swimming. Participants will be encouraged to incorporate exercise into their daily lives and guided to gradually increase their aerobic activity to a moderate intensity of 30 minutes, 5 days a week with blocks of at least 10 minutes being appropriate[42]. Physiotherapists will guide participants to self-monitor their aerobic exercise at a moderate intensity equivalent to at least 'somewhat hard' on the Borg RPE Scale (13-14) and corresponding heart rate, monitored on a wearable heart-rate sensor.

Following 3 months of guided exercise by the physiotherapists, participants will be encouraged to maintain the exercise program over the subsequent 6 months unguided.

Physiotherapists: We will recruit approximately 8 practising musculoskeletal physiotherapists in metropolitan Melbourne to deliver interventions for the trial on Independent Contractor Agreements. The same therapists will deliver intervention to both groups online via Zoom. The intervention will be protocolised. Participants will be randomly allocated to a physiotherapist.

To be eligible to deliver the intervention in this clinical trial, physiotherapists must:

- hold current registration to practice as a physiotherapist;
- have a receptionist at their clinical practice to facilitate patient booking and communication with research staff;
- have an Australian Business Number;
- be willing to undertake trial training requirements (see below);
- be willing and able to deliver interventions between July 2019 and Sept 2022;
- practice around the metropolitan Melbourne area;
- have treated at least five individuals with hip OA in the past 12 months.

Physiotherapist training
Physiotherapists will be required to attend the University of Melbourne for a 4-hour training session. The training will address clinical trial procedures and treatments. Treatment manuals and copies of patient resources (e.g. exercise sheets and information brochures for handouts) will be provided to the physiotherapists in electronic and hard-copy. Physiotherapists will be provided with a Zoom Information Booklet that contains detailed step-by-step instructions on using Zoom. Physiotherapists will also receive personal assistance from the Trial Coordinator to ensure they are comfortable using the technology prior to their first online consult.

Treatment fidelity: Paper-based treatment notes from each consultation will be assessed by research staff for physiotherapist adherence to trial protocols. Fidelity will be recorded as the number and proportion of consultations where the RPE equalled or exceeded 'some-what hard' for aerobic component (i.e. 13-14 on the Borg RPE scale) and 'very hard' for the strengthening program (i.e. 7-8 on the modified Borg scale). The mean (SD) rating of perceived exertion for each participant's exercise program will be determined over their consultations, as recorded in the treatment notes.

9. ADVERSE EVENT REPORTING

Adverse events (AE's) are defined as any untoward medical occurrence in a clinical trial participant that does not necessarily have a causal relationship with the treatment. A related adverse event, or harm, is an adverse event that appears to be caused by the trial interventions.

Serious adverse events (SAE) are defined as any untoward medical occurrence that:

- Results in death,
- Is life-threatening
- Requires hospitalisation or prolongation of existing inpatientś hospitalisation,
- Results in persistent or significant disability or incapacity,
- Is a congenital anomaly or birth defect

Any other important medical condition which, although not included in the above, may require medical or surgical intervention to prevent one of the outcomes listed whether or not it is deemed related to the trial intervention.

A related adverse event will be defined as an adverse event that appears to be caused by the exercise programs. Participants who experience any related adverse events during the first 3 months will be instructed to discuss these with their project physiotherapist who will institute appropriate advice. Any adverse event that the physiotherapist feels unable to manage will be reported by the physiotherapist to the research team via the study email. Any risks to participants are likely to be minor and transient. All related adverse events during the first 3 months will be recorded by physiotherapists in the treatment notes. The study physiotherapist will be asked to return their treatment notes upon completing the 3-month study treatment and the research team will document these related adverse events from the treatment notes into the study adverse events log.

If participants experience any related adverse events as a result of performing the exercise programs unsupervised (3 to 9 months), they will be advised to contact the research team via the study email and a practising physiotherapist from the research team will advise the participant accordingly. The research team will document the related adverse events in the study adverse events log.

Participants will be asked about any non-related adverse events at 3 months and 9 months via a questionnaire. Participants will record the nature of reported adverse events, how long they lasted and what action they took (i.e. taking medication or seeing a health professional). The research team will document the non-related adverse events in the study adverse events log.

Participants will be advised to report any serious adverse events (incapacitating, life-threatening, hospitalisation or death) to the Trial Coordinator as soon as they can by telephone, which will be documented. Any adverse events reported by telephone (by either the participant and/or the physiotherapist) will be reported to the internal trial monitoring committee (The Principal Investigator, the Trial Coordinator and at least one other Investigator who will meet fortnightly to monitor the progress of the trial), who will be responsible for deciding what action, if any, is needed on a case by case basis. Due to the low-risk nature of the interventions in this trial, serious adverse events are extremely unlikely but will be reported to the Sponsor's Ethics Committee should they occur.

Serious adverse events (SAEs) will be reported to the Chief Investigator within 24 hours of the research team becoming aware of them, and the Chief Investigator will determine causality and expectedness. SAEs deemed unexpected and related to the trial will be reported to the research ethics committee within 72 hours using the Serious Adverse Event Form. All participants experiencing serious adverse events will be followed-up as per protocol until the end of the study period (i.e. 9 months). All recorded adverse events will be reported to the Trial Management Committee as blinded data at the monthly meetings.

Related adverse events are expected to be minimal given that previous trials have investigated a combination of aerobic and strengthening exercise in people with hip osteoarthritis and reported no serious adverse events.[43, 44] Adverse events that may be expected as part of the interventions or usual care, which do not need to be reported immediately to the HREC include: transient increase in hip pain, stiffness and swelling or delayed onset muscle soreness due to increased exercise or physical activity. There are no serious adverse events that are expected for the interventions in this study.

9.1 Specific Safety Considerations (E.g. Radiation, Toxicity)

Participants will be exposed to small amounts of radiation through acquisition of the DEXA scans (baseline and follow-up) and hip x-ray (baseline only, and only if participant does not have hip x-ray within past 12 months). As part of everyday living, everyone is exposed to naturally occurring background radiation and receives a dose of about 2 millisievert (mSv) each year. The additional effective dose participants will receive from entering this trial is approximately 0.6mSv. At this dose level, no harmful effects of radiation have been demonstrated as any effect is too small to measure. The risk is believed to be very low.

10. STATISTICAL METHODS

10.1 Sample Size Estimation

Primary endpoints of the trial are between-group differences in change in average overall hip pain intensity and change in physical function (Western Ontario and McMaster Universities Osteoarthritis Index) at 3 months. In OA trials, the minimum clinically important difference is a change in pain of 1.8 units (out of 10) and change physical function of 6 units (out of 68). Based on our recently completed RCT in hip OA, we assume a between-participant standard deviation of 2.2 for pain and 13 units for physical function and a baseline to follow-up correlation of 0.46 for pain and 0.40 for physical function. To achieve 90% power and 5% significance level we require 83 participants per group. Allowing for a 15% loss to follow-up rate (informed by previous hip OA36, 42 trials), we aim to randomise a total of 196 participants over a period of 30 months.

10.2 Population to be analysed

See section 5.2 Eligibility Criteria

10.3 Statistical Analysis Plan

AI-Kasza will analyse the data in a blinded manner. Main comparative analyses between-group will be performed using intention-to-treat. Multiple imputation will be used to account for missing data if >5% of data are missing. For the primary hypothesis, difference in mean change in pain and function (baseline minus follow-up) will be compared between groups using linear regression models adjusting for baseline values, with random effects to account for clustering by therapist. Similar analyses will be conducted for continuous secondary outcomes. Perceived ratings of change will be compared using a logistic regression model with random effect for therapist. A sensitivity analysis will estimate treatment effects assuming full adherence, using a two-stage least squares approach. Effect of patient characteristics on outcomes will be explored by including relevant terms in the models. Standard diagnostic plots will verify model assumptions.

10.4 Interim Analyses

It is not anticipated that any interim analyses will be performed.

11. DATA MANAGEMENT

11.1 Data Collection & Storage

Identifiable data:

- Screening information and study consent forms will be stored within a website (Qualtrics or REDCap) and accessible only by password to the researchers. If participants prefer to complete consent forms in hard copy, paper consent forms will be stored in locked filing cabinets, separate from a cabinet containing any de-identifiable data and only accessible to the researchers.
- Details of people screened will be stored electronically in a Microsoft Access/Excel database, accessible only to the research team and stored securely on password-protected servers.
- X-ray images will be stored electronically at the radiology clinics involved in the study and securely stored and subject to the normal confidentiality guidelines adhered to at each clinic. Researchers will access the x-ray images via log-in to the clinic software and will export de-identified images, labelled with appropriate participant codes and store securely on password protect servers. For participants who have their own eligible x-rays, researchers will create a de-identified electronic copy of the x-ray, label with participant code for storage on password protected servers. We will post back the x-rays to participants who have their own x-rays.
- Cardiorespiratory fitness and body composition data will be stored at the Be Active Sleep and Eat (BASE) Facility, Monash University and securely stored on password-protected servers and subject to the normal confidentiality guidelines adhered at the facility. Researchers will access the password-protected cardiorespiratory fitness and body composition data electronically and export de-identified data, label with appropriate participant codes and store securely on password protected servers.
- Blood samples will be stored at the recommended -70C for up to four years and according to the safe and secure protocols of the Clinical Trials Department at Melbourne Pathology. At the end of the four-year period, Melbourne Pathology will pack and prepare the samples for shipment (frozen, including dry ice), ready to be picked up by the research team. Melbourne Pathology will provide a data extract of the specimens stored in the cryoboxes to the research team in a password protected file. Each extract will be labelled with the appropriate participant codes. The data extract will be stored securely on password protected servers.

Re-identifiable data/coded:

- Questionnaires: may be completed on paper or electronically, and will contain only participant codes, and no identifying information. Paper copies will be stored in locked filing cabinets, separate from a cabinet containing any identifiable data and only accessible to the researchers. Electronic copies will be stored in Qualtrics/REDCap website, accessible only to the researchers by password protection. Data from within Qualtrics/REDCap will be exported to Microsoft Excel and other statistics packages used by the researchers for analyses and stored securely on password-protected servers.
- Laboratory assessments at University of Melbourne: Data will be collected either using pen/paper or online using REDCap into coded data sheets. All computer files will be stored on secure and back-up servers, accessibly only to the researchers using a password. If paper is used, data collection sheets will be stored in locked filing cabinets, separate from a cabinet containing any identifiable data and only accessible to the researchers.
- Daily heart rate data: Data will be collected using a wearable physical activity monitor. Participants will create a new account for their activity monitor using an email of their choice. Each account will be connected to a cloud-based system called Fitabase which enables the research team to access daily heart rate data. Each Fitabase account will be assigned a de-identifiable ID. All heart rate data stored in Fitabase will be subject to the confidentiality guidelines of Small Step Lab LLC and is accessible only to the researchers by password protection. Data will be exported to Microsoft Excel and stored securely on password-protected servers.

11.2 Data Confidentiality

No information which could lead to the identification of a participant will be included in the dissemination of results. Only fully non-identifiable data will be presented when disseminating results. All collected data will also be stored in a locked cabinet throughout the course of the study.

11.3 Study Record Retention

Data will be retained for 15 years consistent with clinical trial recommendations outlined in section 2.1.1 of the National Health and Medical Research Council’s “Australian Code for the Responsible Conduct of Research”.

12. ADMINISTRATIVE ASPECTS

The trial will be prospectively registered (ANZ Clinical Trials Registry) and the protocol published in a peer review journal.

12.1 Independent HREC approval

This study has been submitted by the University of Melbourne Human Research Ethics Committee (HREC), reference number 1953795.1

12.2 Participant reimbursement

All participants who are enrolled in the RCT (i.e. provide informed consent and complete baseline assessment) will receive strengthening equipment at no cost to themselves, to use throughout the trial and keep once participation is complete. In addition, participants who complete the 9-month re-assessment will be given a $50 Coles Myer voucher as a token of appreciation for the considerable time they have invested in the trial (such as the inconvenience of completing questionnaires and log books and other data collection procedures).

12.3 Financial disclosure and conflicts of interest

There are no conflicts of interest to declare.

13. USE OF DATA AND PUBLICATIONS POLICY

We will publish a protocol paper prior to completion of the trial. The main trial will be published in an osteoarthritis or general medical journal.

Statistical code may be made available from Dr Kasza, upon request from individual researchers.

Data may be made available from Dr Hall, upon request from individual researchers.

The results of the trial will also be disseminated through avenues such as conference presentations, professional organisations, media, social media and consumer organisations.

14. REFERENCES

1. Murphy LB, Helmick CG, Schwartz TA, et al. One in four people may develop symptomatic hip osteoarthritis in his or her lifetime. *Osteoarthritis and Cartilage.* 11/01/November 2010 2010;18(11):1372-1379.

2. Cross M, Smith E, Hoy D, et al. *The global burden of hip and knee osteoarthritis: estimates from the Global Burden of Disease 2010 study*: BMJ PUBLISHING GROUP; 2014.

3. Victoria. AaO. Arthritis and Osteoporosis Victoria. A problem worth solving. The rising cost of musculoskeletal conditions in Australia. 2013.

4. Australia A. *Counting the Cost. The current and future burden of arthritis.* 2016.

5. Osteoarthritis NCGC. Care & management in adults. London; . *National Institute for Health and Care Excellence, 2014.* 2014.

6. Moseng T, Dagfinrud H, Smedslund G, Østerås N. The importance of dose in land-based supervised exercise for people with hip osteoarthritis. A systematic review and meta-analysis. *Osteoarthritis and Cartilage.* 10/01/October 2017 2017;25(10):1563-1576.

7. Hall M, Wrigley TV, Kasza J, et al. Cross-sectional association between muscle strength and self-reported physical function in 195 hip osteoarthritis patients; 2017, 2017; United States.

8. Marks R. Comorbid depression and anxiety impact hip osteoarthritis disability. *Disability and Health Journal.* 01/01/January 2009 2009;2(1):27-35.

9. van Dijk GM, Veenhof C, Schellevis F, et al. Comorbidity, limitations in activities and pain in patients with osteoarthritis of the hip or knee. *BMC musculoskeletal disorders.* 2008;9:95.

10. Bieler T, Siersma V, Magnusson SP, Kjaer M, Christensen HE, Beyer N. In hip osteoarthritis, Nordic Walking is superior to strength training and home-based exercise for improving function. *Scandinavian Journal of Medicine & Science in Sports.* 2017;27(8):873-886.

11. Penninx BWJH, Rejeski WJ, Pandya J, et al. Exercise and depressive symptoms: a comparison of aerobic and resistance exercise effects on emotional and physical function in older persons with high and low depressive symptomatology. *The Journals of Gerontology, Series B.* 2002(2):124.

12. Loureiro A, Mills PM, Barrett RS. Muscle weakness in hip osteoarthritis: a systematic review. *Arthritis Care Res (Hoboken).* Mar 2013;65(3):340-352.

13. Dobson F, Hinman RS, Hall M, et al. Reliability and measurement error of the Osteoarthritis Research Society International (OARSI) recommended performance-based tests of physical function in people with hip and knee osteoarthritis. *Osteoarthritis and cartilage.* Nov 2017;25(11):1792-1796.

14. Villareal DT, Aguirre L, Gurney AB, et al. Aerobic or Resistance Exercise, or Both, in Dieting Obese Older Adults. *The New England journal of medicine.* May 18 2017;376(20):1943-1955.

15. Lord SR, Menz HB. Physiologic, psychologic, and health predictors of 6-minute walk performance in older people. *Arch Phys Med Rehabil.*83(7):907-911.

16. Dobson F, Hinman RS, Roos EM, et al. OARSI recommended performance-based tests to assess physical function in people diagnosed with hip or knee osteoarthritis. *Osteoarthritis and cartilage.* Aug 2013;21(8):1042-1052.

17. Hawker GA, Gignac MA, Badley E, et al. A longitudinal study to explain the pain-depression link in older adults with osteoarthritis. *Arthritis care & research.* Oct 2011;63(10):1382-1390.

18. Penedo FJ, Dahn JR. Exercise and well-being: a review of mental and physical health benefits associated with physical activity. *Current opinion in psychiatry.* Mar 2005;18(2):189-193.

19. Bellamy N. Osteoarthritis clinical trials: candidate variables and clinimetric properties. *Journal of Rheumatology.* 1997;24(4):768-778.

20. Bellamy N, Buchanan WW, Goldsmith CH, Campbell J, Stitt LW. Validation study of WOMAC: a health status instrument for measuring clinically important patient relevant outcomes to antirheumatic drug therapy in patients with osteoarthritis of the hip or knee. *The Journal of rheumatology.* Dec 1988;15(12):1833-1840.

21. Osborne RH, Hawthorne G, Lew EA, Gray LC. Quality of life assessment in the community-dwelling elderly: validation of the Assessment of Quality of Life (AQoL) Instrument and comparison with the SF-36. *Journal of Clinical Epidemiology.* Feb 2003;56(2):138-147.

22. McAuley E, Blissmer B, Katula J, Duncan TE. Exercise environment, self-efficacy, and affective responses to acute exercise in older adults. *Psychology & Health.* 2000;15(3):341-355.

23. Newell AM, VanSwearingen JM, Hile E, Brach JS. The Modified Gait Efficacy Scale: Establishing the Psychometric Properties in Older Adults. *Physical Therapy.* 2012;92(2):318-328.

24. Shelby RA, Somers TJ, Keefe FJ, et al. Brief Fear of Movement Scale for osteoarthritis. *Arthritis Care & Research.* 2012;64(6):862-871.

25. Henry JD, Crawford JR. The short-form version of the Depression Anxiety Stress Scales (DASS-21): construct validity and normative data in a large non-clinical sample. *The British journal of clinical psychology.* Jun 2005;44(Pt 2):227-239.

26. Buysse DJ, Reynolds CF, 3rd, Monk TH, Berman SR, Kupfer DJ. The Pittsburgh Sleep Quality Index: a new instrument for psychiatric practice and research. *Psychiatry research.* 1989;28(2):193-213.

27. Belza B, Miyawaki CE, Liu M, et al. A Systematic Review of Studies Using the Multidimensional Assessment of Fatigue Scale. *Journal of nursing measurement.* Apr 1 2018;26(1):36-75.

28. Pua YH, Wrigley TV, Cowan SM, Bennell KL. Intrarater test-retest reliability of hip range of motion and hip muscle strength measurements in persons with hip osteoarthritis. *Arch Phys Med Rehabil.* Jun 2008;89(6):1146-1154.

29. Pua YH, Wrigley TV, Collins M, Cowan SM, Bennell KL. Association of physical performance with muscle strength and hip range of motion in hip osteoarthritis. *Arthritis and rheumatism.* Apr 15 2009;61(4):442-450.

30. Prochilo G, Costa RJS, Hassed C, Chambers R, Molenberghs. The Effects of a 16 week aerobic exercise and mindfulness-based intervention on chronic psychosocial stress: a pilot study. *PsyArXiv.* 2019.

31. Horvath AO, Greenberg LS. Development and validation of the Working Alliance Inventory. *Journal of Counseling Psychology.* 1989;36(2):223-233.

32. Lorig K, Chastain RL, Ung E, Shoor S, Holman HR. Development and evaluation of a scale to measure perceived self-efficacy in people with arthritis. *Arthritis and Rheumatism.* Jan 1989;32(1):37-44.

33. Gardner B, Abraham C, Lally P, de Bruijn G-J. Towards parsimony in habit measurement: Testing the convergent and predictive validity of an automaticity subscale of the Self-Report Habit Index. *International Journal of Behavioral Nutrition and Physical Activity.* 08/01/ 2012;9(1):102-102.

34. Rhodes RE, Rebar AL. Conceptualizing and Defining the Intention Construct for Future Physical Activity Research; 2017, 2017; United States.

35. Sangha O, Stucki G, Liang MH, Fossel AH, Katz JN. The Self-Administered Comorbidity Questionnaire: a new method to assess comorbidity for clinical and health services research. *Arthritis and rheumatism.* Apr 15 2003;49(2):156-163.

36. Kellgren JH, Jeffrey MR, Ball J. *The Epidemiology of Chronic Rheumatism: Atlas of Standard Radiographs.* Vol Volume 2. Oxford: Blackwell Scientific; 1963.

37. Chang WJ, Bennell KL, Hodges PW, et al. Addition of transcranial direct current stimulation to quadriceps strengthening exercise in knee osteoarthritis: A pilot randomised controlled trial. *PloS one.* 2017;12(6):e0180328.

38. Coggon D, Ntani G, Palmer KT, et al. Disabling musculoskeletal pain in working populations: is it the job, the person, or the culture? *Pain.* Jun 2013;154(6):856-863.

39. Freynhagen R, Baron R, Gockel U, Tolle TR. painDETECT: a new screening questionnaire to identify neuropathic components in patients with back pain. *Current medical research and opinion.* Oct 2006;22(10):1911-1920.

40. National Clinical Guideline C. National Institute for Health and Clinical Excellence: Guidance. *Osteoarthritis: Care and Management in Adults*. London: National Institute for Health and Care Excellence (UK)

Copyright © National Clinical Guideline Centre, 2014.; 2014.

41. Riebe D, Franklin BA, Thompson PD, et al. Updating ACSM's Recommendations for Exercise Preparticipation Health Screening. *Medicine and science in sports and exercise.* Nov 2015;47(11):2473-2479.

42. Glazer NL, Lyass A, Esliger DW, et al. Sustained and Shorter Bouts of Physical Activity are Related to Cardiovascular Health. *Medicine and science in sports and exercise.* 2013;45(1):109-115.

43. French HP, Cusack T, Brennan A, et al. Exercise and manual physiotherapy arthritis research trial (EMPART) for osteoarthritis of the hip: a multicenter randomized controlled trial. *Arch Phys Med Rehabil.* Feb 2013;94(2):302-314.

44. Teirlinck CH, Luijsterburg PA, Dekker J, et al. Effectiveness of exercise therapy added to general practitioner care in patients with hip osteoarthritis: a pragmatic randomized controlled trial. *Osteoarthritis and cartilage.* Jan 2016;24(1):82-90.

15. APPENDICES

List of Attachments included:

Title of Form (file name)	Description	Date	Version Number
PHOENIX Advertising Examples.doc	Recruitment advertisements	1/05/2020	1
PHOENIX Consent.docx	Study consent form	1/05/2020	1
PHOENIX Log Book.doc	Participant weekly log book	1/05/2020	1
PHOENIX Phone Screening Form.doc	Checklist used in phone screening of potential participants.	11/10/2020	3
PHOENIX Plain Language Statement.docx	Plain Language Statement	19/05/2021	6
PHOENIX Participant Questionnaire.doc	Study questionnaire – Document includes sections relevant to only the baseline data collection time-point.	11/10/2020	3
PHOENIX GP DEXA Results Letter.docx	DEXA results letter addressed to participant's GP	1/05/2020	1
PHOENIX GP Mood Letter.docx	Mood letter addressed to participant	1/05/2020	1
PHOENIX Participant DEXA Results Letter.docx	DEXA results letter addressed to participant	1/05/2020	1
PHOENIX Online Screen.doc	Checklist used in online screening of potential participants	10/11/2020	3
PHOENIX Results Summary.docx	Results summary letter addressed to participant	1/05/2020	1
PHOENIX Exercise Clearance GP Letter.docx	Participant exercise clearance letter addressed to participant's GP	1/05/2020	1
PHOENIX HRE-Project application_v3.docx	Human research ethics project application form	10/11/2020	5
PHOENIX Body Tissue Genetic Research Module.docx	Body tissue genetic research module	1/05/2020	1
PHOENIX Ionising Radiation Module.pdf	Ionising radiation module (DEXA scan and x-ray)	1/05/2020	1
PHOENIX Medical Physicist report.pdf	Medical physicist report – diagnostic medical physics risk assessment	10/04/2019	1
PHOENIX Serious Adverse Event form.pdf	Serious adverse events form	1/05/2020	1
Participant Zoom Information Booklet	Instructions on how to setup and use Zoom	11/10/2020	2
Physiotherapist Zoom Information Booklet	Instructions on how to setup and use Zoom	11/10/2020	2
