# Supplementary material for: Effects of adding aerobic physical activity to strengthening exercise on hip osteoarthritis symptoms: protocol for the PHOENIX randomised controlled trial
Source: BMC Musculoskelet Disord. 2022 Apr 18;23:361. doi: 10.1186/s12891-022-05282-0 (PMC9014787; doi:10.1186/s12891-022-05282-0)
Supplement: Supplementary file 3 — Additional file 3. PHOENIX Protocol Amendments. [file 12891_2022_5282_MOESM3_ESM.pdf]

Ethics amendment #	Ethics ID	Ethics approval date	Participant Recruitment	Protocol date	Protocol version
Initial ethics approval	1953795.1	7.06.2019	Not yet started		1
Amendment 1	1953795.2	6.09.2019	No yet started	6.09.2019	1
Participant recruitment started on 15.09.2019					
Registration approved 20.09.2019<br>First participant randomised 15.10.2019					
Amendment 2	1953795.3	23.01.2020	In progress	23.01.2020	2
Recruitment paused 17.03.2021 due to Covid-19					
Amendment 3	1953795.4	19.03.2020	Paused	19.03.2020	3
Changes made to protocol and registration. We did not submit an amendment; these changes were submitted in amendment 4.			Paused	30.06.2020	4
Amendment 4	1953795.5	21.10.2020	Paused	10.11.2020	5
Participant recruitment resumed 14.11.2020					
Amendment 5	12710 (2.2)	19.05.2021	In progress	19.05.2021	6
Amendment 6	12710	8.12.2021	In progress	8.12.2021	7
Amendment 7	12710	16.12.2021	In progress	8.12.2021	7

Amendment number	Amendment described
Amendment 2	**Outcomes for nested study added**<br>• We have added eight items to the self-report participant questionnaire at baseline, 6-week, 3-month and 9-month timepoints. These items will ask participants about behaviour change and their motivation for exercise.<br>• The Overall Satisfaction with Exercise Program self-reported question has been added to the 6-week participant questionnaire.<br>**Addition of two names researchers**<br>• Ms Brook Brisbine<br>• Mr Alexander Kimp
Amendment 3	**Physiotherapy consultations**<br>• Participants will attend up to 9 physiotherapist consultations, preferably face-to-face. If face-to-face consultations are not advisable (e.g. COVID-19), or not possible (e.g. unable to travel), consultations will be conducted online via Zoom.

	**Research Design & Method**<br>• Participants will attend appointments preferably face-to-face. However, if face-to-face consultations are not advisable (e.g. COVID-19), or not possible (e.g. unable to travel), consultations will be conducted online via Zoom.
Amendment 4	**Inclusion criteria**<br>• Replaced ACR inclusion criteria with NICE inclusion criteria.<br>• New inclusion criteria: Access to a device with internet connection<br>**Removed 3 exclusion criteria**<br>• Hip pain symptoms associated with extra-articular or lumbar pathology<br>• Any other joint or muscle problem, such as back, other hip, knees, ankles or feet, which is worse than hip pain on affected side<br>• Performed exercise prescribed by health professional specifically for the hip or lower back in the past 6 months<br>**Participant tasks/Secondary Outcome Data Collection**<br>• We will not acquire data of 13 secondary outcomes that require face-to-face contact whilst government and university restrictions are in place. Participants will not attend baseline and follow up testing appointments at the University of Melbourne or Monash BASE. Participants will not provide baseline and follow up blood samples at Melbourne Pathology or attend x-ray appointments at baseline.<br>**Participant tasks/Physiotherapy Consultations**<br>• Permanently deliver all physiotherapy treatments online (previously granted approval 1953795.4). Participants will attend their 9 physiotherapy consultations online via videoconference (Zoom) rather than visiting the clinic and taking part in a face-to-face consult.<br>**Secondary outcome: height and weight**<br>• Participants will be asked to self-report their height and weight in the baseline questionnaire if face-to-face appointments are not permitted by the University of Melbourne.<br>**Randomisation schedule**<br>• A new randomisation schedule will be created to include random allocation of a study physiotherapist to a participant. Participants will no longer be choosing their physiotherapist based on location of the clinic.
Amendment 5	**Blood sample storage:**<br>• The blood samples will and have been stored at the recommended -70C for up to 4 years and according to the safe and secure protocols of the Clinical Trials Department at Melbourne Pathology. At the end of the four year period, Melbourne Pathology will pack and prepare the samples for shipment (frozen, including dry ice), ready to be picked up by the research team. Melbourne Pathology will provide a data extract of the specimens stored in the cryoboxes to the research team in a password protected file. Each extract will be labelled with appropriate participant codes. The data extract will be stored securely on password protected servers.<br>**Daily heart rate data**<br>• Instead of providing participants with a non-identifiable email address to create an activity monitor account, participants will create a new account using an email of their choice. Each account will still be assigned a de-

	identifiable ID when connected to cloud-based system, Fitabase. However, the ID assigned will not be randomly generate via Qualtrics. We will instead assign the participant's study ID.<br>**Secondary outcome**<br>• Added Patient Specific Functional Scale to in ethics application and listed as a secondary outcome (it was previously only listed in participant questionnaire).
Amendment 6	**Addition of six named researchers**<br>a. Ms Fiona McManus has joined the team and will replace Dr Jessica Kasza as biostatistician.<br>b. Dr Karen Lamb has joined the team and will support and guide Ms Fiona McManus (biostatistician) with the statistics for the PHOENIX study. Dr Lamb will provide oversight during the course of the analysis and reporting.<br>c. A/Prof Ricardo Da Costa will contribute to the collection and analysis of dual-energy x-ray absorptiometry (DEXA) data and cardiorespiratory fitness data.<br>d. Dr Melanie Plinsinga specialises in persistent, musculoskeletal pain, particularly in osteoarthritis. Dr Plinsinga is involved as a pain specialist and will contribute to the analysis of the quantitative sensory tests.<br>e. Dr David Klyne will contribute to the selection of biomarkers for analysis, methods for analysis and interpretation.<br>f. Dr Nick Murphy is an orthopaedic registrar at Central Coast Local Health District in NSW. He brings expertise in imaging and will grade the hip x-rays.
Amendment 7	**Updated the Pain Language Statement and Consent Form to reflect changes made in Amendment 6.**
