# Supplementary material for: Effects of adding aerobic physical activity to strengthening exercise on hip osteoarthritis symptoms: protocol for the PHOENIX randomised controlled trial
Source: BMC Musculoskelet Disord. 2022 Apr 18;23:361. doi: 10.1186/s12891-022-05282-0 (PMC9014787; doi:10.1186/s12891-022-05282-0)
Supplement: Supplementary file 4 — Additional file 4. PHOENIX Consent. [file 12891_2022_5282_MOESM4_ESM.pdf]

Consent Form

Centre for Health, Exercise and Sports Medicine
Department of Physiotherapy, School of Health Sciences

Title: Exercise for people with hip osteoarthritis – the PHOENIX study

Primary researcher: Dr Michelle Hall
Additional researchers: Dr Kim Allison, Prof Rana Hinman, Ms Libby Spiers, Ms Gabrielle Knox, Prof Kim Bennell, Dr Fiona Dobson, Ms Fiona McManus, Dr Karen Lamb, A/Prof Ricardo Da Costa, Dr Melanie Plinsinga, Dr David Klyne, Dr Nick Murphy

1. I consent to participate in this study, the details of which have been explained to me, and I have been provided with a written plain language statement to keep.

2. I understand that after I sign and return this consent form it will be retained by the researcher.

3. I understand what my participation will involve, and I agree that the researcher may use the results as described in the plain language statement.

4. I acknowledge that:

(a) the possible effects of participating in the program have been explained to my satisfaction;

(b) I have been informed that I am free to withdraw from the project at any time without explanation or prejudice and to withdraw any unprocessed data I have provided;

(c) the study is for the purpose of research;

(d) I have been informed that the confidentiality of the information I provide will be safeguarded subject to any legal requirements;

(e) I wish to receive a copy of the summary project report of research findings at the end of the study ☐ yes ☐ no (please tick)

(f) I agree to opt-in and allow researchers to access my daily heart rate data over the first 3 months of the study which will be stored by Fitabase (Small Steps Labs). ☐ yes ☐ no (please tick)

(g) I agree to opt-in to have a blood test at the beginning of the study and 3 months later, which will be stored by Melbourne Pathology for up to 4 years and analysed to look at inflammatory markers in my blood. ☐ yes ☐ no (please tick)

I, ______________________________ consent to participate in the above study.

Participant signature ________________________ Date ____________
