# Supplementary material for: Effects of adding aerobic physical activity to strengthening exercise on hip osteoarthritis symptoms: protocol for the PHOENIX randomised controlled trial
Source: BMC Musculoskelet Disord. 2022 Apr 18;23:361. doi: 10.1186/s12891-022-05282-0 (PMC9014787; doi:10.1186/s12891-022-05282-0)
Supplement: Supplementary file 5 — Additional file 5. PHOENIX Strengthening Exercises. [file 12891_2022_5282_MOESM5_ESM.pdf]

Home based strengthening exercise protocol

Minimum of 4 and maximum of 6 exercises, with progression as appropriate:<br>1 Hip extensor exercise<br>1 Hip flexor exercise<br>1 Hip abductor exercise<br>1 Functional/knee extensor exercise<br>Once a participant is able to do more than the minimum of 4 exercises, an exercise will be added from the hip adductor group or an additional exercise from the above groups.			
**Hip Extensors**			
Leg lift	Non-weight bearing	Face-down leg lift	**Instruction:** Lie on stomach with hands folded under your chin. Bend the knee of your study hip, with toes pulled up. Push heel towards ceiling, lifting leg off the bed and squeezing buttocks the whole time. Hold for 1-2 seconds, then lower leg.
	Non-weight bearing	4-point-kneel leg lift	**Instruction:** On all fours, slowly kick the study leg backwards, squeezing buttocks and maintain 90 degrees bend in the knee. Bring leg back in and repeat.<br>**Progression:** Straighten leg of study hip; add ankle cuff weight; increase exercise band resistance (yellow through to blue).
Bridging	Weight bearing	Bridge	**Instruction:** Lie on a firm surface with knees bent and feet flat on surface. Place feet hip-width apart. Lift buttocks and hips from the surface. Hold for 2-3 second then slowly lower.<br>**Progression options:** Add weight.
	Weight bearing	Split leg bridge	**Instruction:** Lie on a firm surface. Place feet hip-width apart. Move study leg closer to buttocks and towards centre. Lift buttocks and hips, taking more weight through study leg. Hold for 2-3 seconds then slowly lower.

	Weight bearing	Double to single leg bridge	**Instruction:** Lie on a firm surface with knees bent and feet flat on surface. Squeeze buttocks and lift hips and buttocks from surface. Keep hips level, lift non-study leg off surface. Hold for 2-3 seconds. Ground non-study leg and slowly lower buttocks onto support surface.<br>**Progression options:** Increase duration of the hold; move onto advance single-leg bridge.
	Weight bearing	Single-leg Bridge	Lie on a firm surface with knees bent and feet flat on surface. Lift study leg off the surface. Keeping hips level, use one leg to lift your bottom and pelvis up off the surface. Hold for 2-3 seconds, then slowly lower.
**Functional / Knee Extensors**			
	Weight bearing	Partial squats	**Instruction:** Standing with feet shoulder-width apart. Bend hips and knees. Lower down slightly. Hold for 2-3 seconds and slowly straighten back up.
	Weight bearing	Partial squats against wall	**Instruction:** Lean back against wall and step feet hip-width apart, about 40 cm away from the wall. Slide down wall, stopping before knees go past toes. Hold for 2-3 seconds, and slowly slide back up.<br>**Progression:** Add resistance band around knees; half-way holds (hold for 3 seconds at the halfway point while going up and/or down).
	Weight bearing	Split leg wall squats	**Instruction:** Lean back against wall and step feet hip-width apart, about 40 cm away from the wall. Move your non-study leg a further 15 cm away from the wall. Slide down wall, stopping before knees go past toes. Hold for 2-3 seconds, and slowly slide back up.

	Weight bearing	Sit to stand	**Instruction:** Sit on a chair with feet shoulder-width apart. Slowly stand until fully straight and sit down slowly.<br>**Progression:** Add a resistance band around your knees; half-way holds (hold for 3 seconds at the halfway point while going up and/or down); use a lower chair; hold a weight.
	Weight bearing	Sit to stand with more weight on study leg	**Instruction:** Sit on a chair with feet shoulder-width apart. Take more weight on study leg by either (a) placing non-study leg further forward so that study leg is closer, or (b) shifting both feet sideways so study leg is lined up with middle of body. Push through the heel of study leg and stand up without using hands. Slowly sit down.
	Weight bearing	Step ups	**Instruction:** Place study leg on a step. Push through the heel of study leg and bring up other leg. Lightly touch the non-study foot onto the step, then step it back down to the starting position.<br>**Progression:** Use higher step; hold a weight.
**Hip abductor exercises**			
	Weight bearing	Standing side leg raises	**Instruction:** Standing with resistance band around ankles. Lift study leg out to side, leading with heel. Hold for 1-3 seconds then lower slowly.<br>**Progression:** Increase exercise band resistance (yellow through to blue).
	Weight bearing	Standing leg wall press	**Instruction:** Stand sideways with non-study leg against a wall. Lift the non-study leg off the floor, so that hip, thigh and knee touch the wall. Keep body still and push non-study leg into the wall. Hold 3-5 seconds then lower slowly.

	Non-weight bearing	Side lying leg raises	**Instruction:** Lie on a firm surface with study leg on top. Bend bottom leg and use arms for support. Slowly raise study leg, keeping knee faceting forwards and avoid rolling backwards. Hold for 1-3 seconds then lower slowly.<br>**Progression** Add cuff weight on the top leg.
	Weight bearing	Crab walking	**Instruction:** Place resistance band around thighs/knees, and separate legs about 10 cm. Slight bend both knees. Step sideways against the tension of the resistance band, keeping legs apart. Take 3 steps right and 3 steps left. Continue for 30 seconds to complete 1 round.<br>**Progression:** Place resistance bands around ankles; incorporate zig-zags; increase exercise band resistance (yellow through to blue).
**Hip flexor exercises**			
	Non-weight bearing	Crook lying hip bends	**Instruction:** Lie on a firm surface with knees bent and feet flat on the floor. Keeping your non-study leg bent, slowly raise the study leg into the air. Slowly lower down again.<br>**Progression:** Add weight to the study leg (just above the knee); straighten the knee.
	Non-weight bearing	Face up lying hip bends off edge of the bed	**Instruction:** Lie on a firm surface, with your study leg hanging off the end of the bed. Bring the knee of the non-study leg to chest and hold with arms. Tuck your tail under and keep your back flat against the bed. Slowly raise your study leg into the air keeping the knee bent. Slowly lower study leg back down to bed level, no further.<br>**Progression:** Add weight to the study leg(just above the knee); straighten the knee.

	Weight bearing	Standing knee raises	**Instruction:** Stand with feet shoulder-width apart. Use a table or chair for balance. Bend study leg up so that thigh is parallel with the ground. Slowly lower back down. Touch foot lightly with the ground before repeating.
**Hip adductor strengthening exercises**			
	Non-weight bearing	Crook lying leg squeeze with 5 second hold	**Instruction:** Lie on a firm surface with knee bent and feet on a firm surface. Place a ball or cushion between knees. Keep tail tucked under. Gently squeeze knees together and build to a moderate pressure. Hold for 5 seconds, then relax.
	Weight bearing	Standing resistance band adduction	**Instruction:** Tie a resistance band to a stable support and loop the band around the study leg ankle. Step away from the support to create tension in the band. Move your study leg to the midline, pulling against the resistance bank. Keep all your weight supported on your non-study leg. Slowly return your study leg to the starting position. **Progression option:** Increase resistance (yellow through to blue).
	Non-weight bearing	Side lying hip adduction	**Instruction:** Lie on your side with the non-study leg on top. Place your non-study foot in front of other knee. Straighten study leg, with knee facing forward. Keep body still and slowly life the study leg up and off the bed/floor. Slowly lower back down. **Progression:** Add weight to the study leg.
